# Genetic diversity and population structure of *Musa* accessions in *ex situ* conservation

**DOI:** 10.1186/1471-2229-13-41

**Published:** 2013-03-12

**Authors:** Onildo Nunes de Jesus, Sebastião de Oliveira e Silva, Edson Perito Amorim, Claudia Fortes Ferreira, José Marcello Salabert de Campos, Gabriela de Gaspari Silva, Antonio Figueira

**Affiliations:** 1 Centro de Energia Nuclear na Agricultura, Universidade de São Paulo, Av. Centenário, 303, CP 96, Piracicaba, SP, 13400-970, Brazil; 2 Escola Superior de Agricultura “Luiz de Queiroz”, Universidade de São Paulo, Av. Pádua Dias, 11, Piracicaba, SP, 13418-900, Brazil; 3 EMBRAPA Mandioca Fruticultura, R. Embrapa s/n, Cruz das Almas, BA, 44380-000, Brazil; 4 Instituto de Ciências Biológicas, Universidade Federal de Juiz de Fora, Campus Martelos, Juiz de Fora, MG, 36016-900, Brazil

**Keywords:** Association mapping, Banana, Evolution, Flow cytometry, Internal transcribed spacer, Microsatellite, Simple sequence repeat, Structure

## Abstract

**Background:**

Banana cultivars are mostly derived from hybridization between wild diploid subspecies of *Musa acuminata* (A genome) and *M. balbisiana* (B genome), and they exhibit various levels of ploidy and genomic constitution. The Embrapa *ex situ Musa* collection contains over 220 accessions, of which only a few have been genetically characterized. Knowledge regarding the genetic relationships and diversity between modern cultivars and wild relatives would assist in conservation and breeding strategies. Our objectives were to determine the genomic constitution based on Internal Transcribed Spacer (ITS) regions polymorphism and the ploidy of all accessions by flow cytometry and to investigate the population structure of the collection using Simple Sequence Repeat (SSR) loci as co-dominant markers based on Structure software, not previously performed in *Musa*.

**Results:**

From the 221 accessions analyzed by flow cytometry, the correct ploidy was confirmed or established for 212 (95.9%), whereas digestion of the ITS region confirmed the genomic constitution of 209 (94.6%). Neighbor-joining clustering analysis derived from SSR binary data allowed the detection of two major groups, essentially distinguished by the presence or absence of the B genome, while subgroups were formed according to the genomic composition and commercial classification. The co-dominant nature of SSR was explored to analyze the structure of the population based on a Bayesian approach, detecting 21 subpopulations. Most of the subpopulations were in agreement with the clustering analysis.

**Conclusions:**

The data generated by flow cytometry, ITS and SSR supported the hypothesis about the occurrence of homeologue recombination between A and B genomes, leading to discrepancies in the number of sets or portions from each parental genome. These phenomenons have been largely disregarded in the evolution of banana, as the “single-step domestication” hypothesis had long predominated. These findings will have an impact in future breeding approaches. Structure analysis enabled the efficient detection of ancestry of recently developed tetraploid hybrids by breeding programs, and for some triploids. However, for the main commercial subgroups, Structure appeared to be less efficient to detect the ancestry in diploid groups, possibly due to sampling restrictions. The possibility of inferring the membership among accessions to correct the effects of genetic structure opens possibilities for its use in marker-assisted selection by association mapping.

## Background

Cultivated bananas and plantains (*Musa* spp.) originated in Southeast Asia and the Western Pacific [1,2]. From the center of origin, *Musa* spp. was introduced into Africa in ancient times and taken by European explorers to the Americas and other parts of the world [3,4]. Currently, bananas and plantains (hereafter jointly called bananas) are widely cultivated in tropical and subtropical regions as important staple foods and commodities in many countries [5].

The large majority of banana cultivars are derived from natural crosses between wild seeded diploid subspecies of *M. acuminata* Colla (A genome) and *M. balbisiana* Colla (B genome) [6]. Most of modern cultivars contains genome combinations with various levels of ploidy, such as diploid (AA; BB; or AB; 2*n* = 2*x* = 22); triploid (AAA; AAB; or ABB; 2*n* = 3*x* = 33); and tetraploid (AAAA; AAAB; AABB; or ABBB; 2*n* = 4*x* = 44) [6]. It is not well established how wild bananas became domesticated, but it is possible that the accumulation of sterility and acquisition of parthenocarpy with the increase of pulp mass and the absence of seeds, followed by human selection, gave rise to the modern predominantly sterile cultivars [7-10].

There are a limited number of *ex situ* conservation collections in the world (http://www.crop-diversity.org/banana/) and even fewer breeding programs associated with an important collection. One of these rare examples is the germplasm collection maintained at ‘Embrapa Mandioca Fruticultura’ Center, located at Cruz das Almas, Bahia, Brazil (12°39'59"S; 39°06'00"W). This *ex situ* collection, with over 220 individual accessions, is derived from the efforts begun in 1981 by the late Dr. Kenneth Shepherd, who used his significant personal networking and credibility with international organizations to obtain and introduce *Musa* spp. germplasm from various countries [11]. Despite the fact that a wide range of genetic resources is maintained, only a few accessions have been used in the breeding program, possibly because of the lack of characterization and genetic identity.

The precise determination of the ploidy and genomic composition of the accessions are of great interest to define hybridization programs, as the combination of these two genomes (A and B) defines the agronomical attributes (for e.g., yield; resistance to biotic factors) as well as the fruit flavor and quality of the resulting hybrid plants [12-14]. In addition, estimation of genetic diversity and genetic relationships among the various wild and cultivated accessions will help to develop novel approaches for breeding and assist long-term conservation strategies.

To determine ploidy in *Musa* spp., chromosome counting [15], estimation of the stomata size and density, or measurement of the pollen grain sizes have been employed [16], whereas for the characterization of the genomic composition (genome A and/or B), a set of 15 standard morphological descriptors have been traditionally used [6]. However, these conventional methods are imprecise, suffering from large environmental effects, and they are tedious and time-consuming, and not applicable on a large scale. Flow cytometry is a quick method that is able to detect small variations in DNA content and efficient for determining ploidy level in *Musa* spp. [17-19]. To determine the genomic composition of the *Musa* genus, PCR-RFLP markers based on the rDNA region developed by Nwakanma et al. [20] appeared to be effective [21], but the results are limited in terms of the ability to estimate the genetic diversity. On the other hand, simple sequence repeat (SSR) loci with genome-specific alleles [22,23] offer the possibility to identify genomic composition and to estimate the genetic diversity and relationships among accessions from an *ex situ* conservation collection.

Despite the multiallelic and highly informative nature of microsatellite (SSR) loci, the allelic information in *Musa* had usually been converted into binary data due to the difficulty in establishing allelic relations between heterozygous genotypes with distinct levels of ploidy [9,21,22,24-29] and polysomic inheritance [29]. The exploration of the co-dominant nature of SSR loci using Bayesian models implemented using the software Structure [30-32] might enable new perspectives of establishing allelic relationships between various accessions to infer about ancestry between cultivars and wild accessions and *M. acuminata* subspecies. The determination of the genetic structure in *ex situ* collections is important to determine the genetic relationships [11,33] and to establish core collections [34]. Further, the use of Structure would enable the estimation of a membership matrix among the accessions, adopted in association mapping models [35] to correct the genetic structuring that leads to false associations (false positives). Association mapping is an approach particularly well suited for *Musa* spp., because non-related individuals can be sampled in a population, such as an *ex situ* germplasm collection or collections of elite varieties [36-38], without the requirement to develop segregating populations, limited in *Musa* by sterility, incompatibility [39], low viability of the hybrids due to chromosomal aberrations, and segregation of unviable gene alleles [40,41].

Therefore, the objectives of this study were (i) to characterize the accessions of the *ex situ* conservation collection in Brazil regarding ploidy and the genomic constitution by flow cytometry and PCR-RFLP; and (ii) to establish the genetic relationships by exploring the co-dominant nature of the SSR loci using the Bayesian model implemented on Structure.

## Methods

### Plant material

A total of 224 accessions of the *Musa* genus were analyzed, including wild and cultivated materials with apparent diverse ploidy and genomic constitution (Table 1; Additional file 1: Table S1). The only passport information available was the origin of the accessions, with a presumed genomic constitution. Classification of the banana accessions as members of subgroups (such as ‘Pome’; ‘Silk’; and ‘Cavendish’) had previously been performed by breeders. Other information, such the subspecies or subgroup, was obtained from the *Musa* Germplasm Information System (http://www.crop-diversity.org/banana/) [42].

**Table 1:** ***Musa *accessions from the *ex situ *collection of ‘Embrapa Mandioca Fruticultura’ Center (Cruz das Almas, Brazil) with information from passport data, and characterization of genomic constitution by flow cytometry, PCR-RFLP of internal transcribed spacers (ITS) regions, and Simple Sequence Region (SSR) loci**

**No**	**Acession**		**Cytometry**		**Molecular markers**				
						**ITS fragments (bp)**				
		**Genome**^**z**^	**2C pg**	**Ploidy**	**530**	**350**	**180**	**Genome**	**SSR**^**v**^	
1	*Musa basjoo*	ES	1.23	2x	++	++		-	-
2	Piraí	BB	1.22	2x		++	++	BB	BB
3	Butuhan	BB	1.23	2x		++	++	BB	BB
4	BB Panama	BB	1.30	2x		++	++	BB	BB
5	Balbisiana França	BB	1.27	2x		++	++	BB	BB
6	Musa Balbisiana	BB	1.25	2x		++	++	BB	BB
7	TIP	ABB	1.98	3x	++	+	+	AAB	AAB
8	Saba Honduras	ABB	1.93	3x	++	++	++	ABB	ABB
9	Saba	ABB	1.95	3x	++	++	++	ABB	ABB
10	Prata Zulu	ABB	M^x^	?^W^	++	+	+	ABB/AAB	ABB
11	Poteau Nain	ABB	M	?	++	+	+	ABB/AAB	ABB
12	Pelipita	ABB	1.89	3x	++	++	++	ABB	ABB
13	Namwa Khom	ABB	1.91	3x	++	++	++	ABB	ABB
14	Namwa Daeng	ABB	1.94	3x	++	++	++	ABB	ABB
15	Muisa Tia	ABB	1.90	3x	++	++	++	ABB	ABB
16	Monthan	ABB	1.93	3x	++	++	++	ABB	ABB
17	Ice Cream	ABB	1.94	3x	++	++	++	ABB	ABB
18	Ice Cream	ABB	1.93	3x	++	++	++	ABB	ABB
19	Gia Hui	ABB	1.90	3x	++	++	++	ABB	ABB
20	Figo Cinza	ABB	1.86	3x	++	++	++	ABB	ABB
21	Espermo	ABB	M	?	++	++	++	ABB	ABB
22	Champa Madras	ABB	1.98	3x	++	++	++	ABB	ABB
23	Cachaco	ABB	1.93	3x	++	++	++	ABB	ABB
24	Cacambou Naine	ABB	1.93	3x	++	++	++	ABB	ABB
25	Bendetta	ABB	1.93	3x	++	++	++	ABB	ABB
26	Abuperak	ABB	1.94	3x	++	++	++	ABB	ABB
27	IAC	AB(H)	1.26	2x	++	+	+	AB	?
28	Yangambi nº 2	AAB	1.93	3x	++			AAA	AAB
29	Warik	AAB	1.94	3x	++	+	+	AAB	AAB
30	Walha	AAB	1.95	3x	++	+	+	AAB	AAB
31	Ustrali	AAB	1.93	3x	++	+	+	AAB	AAB
32	Umpako	AAB	1.89	3x	++	+	+	AAB	AAB
33	Thap Maeo	AAB	1.94	3x	++	+	+	AAB	AAB
34	Trois Vert	AAB	1.90	3x	++	+	+	AAB	AAB
35	Tomnam	AAB	1.94	3x	++	+	+	AAB	AAB
36	Tipo Velhaca	AAB	1.92	3x	++	+	+	AAB	AAB
37	Tip Kham	AAB	1.87	3x	++	+	+	AAB	AAB
38	Thong Ruong	AAB	1.98	3x	++	+	+	AAB	AAB
39	Terrinha	AAB	1.94	3x	++	+	+	AAB	AAB
40	Terra S/ Nome	AAB	1.94	3x	++	+	+	AAB	AAB
41	Tai	ABB	1.99	3x	++	++	++	ABB	ABB
42	Sempre Verde	AAB	1.94	3x	++	+	+	AAB	AAB
43	Saney	AAB	1.94	3x	++	+	+	AAB	AAB
44	Samurá B	AAB	1.89	3x	++	+	+	AAB	AAB
45	Red Yade	AAB	1.95	3x	++	+	+	AAB	AAB
46	Pulut	AAB	1.94	3x	++	+	+	AAB	AAB
47	Pratão	AAB	1.95	3x	++	+	+	AAB	AAB
48	Prata Sta. Maria	AAB	1.94	3x	++	+	+	AAB	AAB
49	Prata P. Aparada	AAB	1.95	3x	++	+	+	AAB	AAB
50	Prata Maceió	AAB	1.93	3x	++	+	+	AAB	AAB
51	Prata Comum	AAB	1.90	3x	++	+	+	AAB	AAB
52	Prata IAC	AAB	1.99	3x	++	+	+	AAB	AAB
53	Prata Anã	AAB	1.94	3x	++	+	+	AAB	AAB
54	Prata Branca	AAB	2.29	4x	++	+	+	AAAB	AAB
55	Poovan	AAB	1.94	3x	++	+	+	AAB	AAB
56	Plantain N. 2	AAB	?	?	++	+	+	AB?	AAB
57	Pinha	AAB	1.94	3x	++	+	+	AAB	AAB
58	Padath	AAB	1.95	3x	++	+	+	AAB	AAB
59	Pacovan	AAB	1.93	3x	++	+	+	AAB	AAB
60	N. 113	AAB	1.89	3x	++	+	+	AAB	AAB
61	Mysore	AAB	1.95	3x	++	+	+	AAB	AAB
62	Muracho	AAB	1.95	3x	++	+	+	AAB	AAB
63	Mongolo	AAB	1.98	3x	++	+	+	AAB	AAB
64	Moenang	AAB	1.99	3x	++	+	+	AAB	AAB
65	Maçã Caule Roxo	AAB	1.90	3x	++	+	+	AAB	AAB
66	Kune	AAB	1.92	3x	++	+	+	AAB	AAB
67	Kingala N.1	AAB	1.95	3x	++	+	+	AAB	AAB
68	Kepok Bung	AAB	1.95	3x	++	+	+	ABB/AAB	ABB/AAB
69	Kelat	AAB	1.87	3x	++	+	+	AAB	AAB
70	Java IAC	AAB	1.99	3x	++	+	+	AAB	AAB
71	Garoto	AAB	1.93	3x	++	+	+	AAB	AAB
72	Figue Rose Naine	AAB	1.93	3x	++			AAA	AAA
73	Eslesno	AAB	1.95	3x	++	+	+	AAB	AAB
74	Curare Enano	AAB	1.99	3x	++	+	+	AAB	AAB
75	Comprida	AAB	1.98	3x	++	+	+	AAB	AAB
76	Chifre De Vaca	AAB	1.92	3x	++	+	+	AAB	AAB
77	Adimoo	AAB	1.93	3x	++	+	+	AAB	AAB
78	AAB S/Nome	AAB	1.95	3x	++	+	+	AAB	AAB
79	BRS Tropical	AAAB	2.50	4x	++			AAAA	AAAB
80	Preciosa	AAAB	1.94	3x	++	+	+	AAB	AAAB
81	Porp	AAAB	2.43	4x	++			AAAB	AAAB
82	Platina	AAAB	2.56	4x	++	+	+	AAAB	AAAB
83	Pacova Ken	AAAB	2.45	4x	++	+	+	AAAB	AAAB
84	BRS Platina	AAAB	2.45	4x	++	+	+	AAAB	AAAB
85	Ouro Da Mata	AAAB	2.46	4x	++	+	+	AAAB	AAAB
86	Ngern	AAAB	2.55	4x	++	+	+	AAAB	AAAB
87	Langka	AAAB	2.48	4x	++	+	+	AAAB	AAAB
88	Garantida	AAAB	2.48	4x	++	+	+	AAAB	AAAB
89	FHIA-21	AAAB	2.49	4x	++	+	+	AAAB	AAAB
90	FHIA-18	AAAB	2.48	4x	++	+	+	AAAB	AAAB
91	FHIA-02	AAAB/AAAA	2.40	4x	++	+	+	AAAB	AAAB
92	FHIA-01	AAAB	2.49	4x	++	+	+	AAAB	AAAB
93	IC - 2	AAAA	2.49	4x	++			AAAA	AAAA
94	Calypso	AAAA	2.43	4x	++			AAAA	AAAA
95	Buccaneer	AAAA	2.45	4x	++			AAAA	AAAA
96	Ambrosia	AAAA	2.47	4x	++			AAAA	AAAA
97	Yangambi Km 5	AAA	1.92	3x	++			AAA	AAA
98	Wasolay	AAA	1.92	3x	++			AAA	AAA
99	Walebo	AAA	1.98	3x	++			AAA	AAA
100	Valery	AAA	1.98	3x	++			AAA	AAA
101	Umbuk	AAA	1.95	3x	++			AAA	AAA
102	Tugoomomboo	AAA	?	?	++	++	++	ABB	AAB
103	Caipira	AAA	1.98	3x	++			AAA	AAA
104	Towoolee	AAA	1.90	3x	++			AAA	AAA
105	Torp	AAA	1.90	3x	++			AAA	AAA
106	Sri	AAA	1.93	3x	++			AAA	AAA
107	Sapon	AAA	1.93	3x	++			AAA	AAA
108	São Tomé	AAA	1.92	3x	++			AAA	AAA
109	Roombum	AAA	1.92	3x	++			AAA	AAA
110	Poyo	AAA	1.91	3x	++			AAA	AAA
111	Pirua	AAA	1.91	3x	++			AAA	AAA
112	Pagatow	AAA	1.91	3x	++			AAA	AAA
113	Ouro Mel	AAA	1.92	3x	++			AAA	AAA
114	Orotawa	AAA	1.93	3x	++			AAA	AAA
115	Nanicão	AAA	1.94	3x	++			AAA	AAA
116	Nam	AAA	1.94	3x	++			AAA	AAA
117	Muga	AAA	M	?	++			A?	AAA
118	Morong	AAA	1.92	3x	++			AAA	AAA
119	Markatooa	AAA	1.91	3x	++			AAA	AAA
120	Maida	AAA	1.94	3x	++			AAA	AAA
121	Leite	AAA	1.93	3x	++			AAA	AAA
122	Lacatan	AAA	1.90	3x	++			AAA	AAA
123	Azedinha	AAB	2.36	4x	++	+	+	AAAB	AAAB
124	Imperial	AAA	1.93	3x	++			AAA	AAA
125	Highgate	AAA	1.91	3x	++			AAA	AAA
126	Gros Michel	AAA	1.94	3x	++			AAA	AAA
127	Grande Naine	AAA	1.92	3x	++			AAA	AAA
128	Dois Cachos	AAA	1.91	3x	++			AAA	AAA
129	Dodoga	AAA	1.94	3x	++			AAA	AAA
130	Cocos	AAA	1.95	3x	++			AAA	AAA
131	Caru Verde	AAA	1.96	3x	++			AAA	AAA
132	Caru Roxa	AAA	1.95	3x	++			AAA	AAA
133	Canela	AAA	1.94	3x	++			AAA	AAA
134	Bakar	AAA	1.91	3x	++			AAA	AAA
135	Bagul	AAA	1.93	3x	++			AAA	AAA
136	Amritsagar	AAA	1.93	3x	++			AAA	AAA
137	Ambei	AAA	1.94	3x	++			AAA	AAA
138	AAA Desconhecida	AAA	1.93	3x	++			AAA	AAA/AAB
139	Zebrinha	AA(W)	1.23	2x	++			AA	AA
140	Selangor	AA(W)	1.25	2x	++			AA	AA
141	Perak	AA(W)	1.26	2x	++			AA	AA
142	Pa Songkla	AA(W)	1.23	2x	++			AA	AA
143	Pa Rayong	AA(W)	1.26	2x	++			AA	AA
144	Pa Phatthalung	AA(W)	1.23	2x	++			AA	AA
145	Pa Musore 3	AA(W)	1.25	2x	++			AA	AA
146	Pa Musore 2	AA(W)	1.27	2x	++			AA	AA
147	Pa Abissinea	AA(W)	1.28	2x	++			AA	AA
148	N.118	AA(W)	1.29	2x	++			AA	AA
149	Monyet	AA(W)	1.23	2x	++			AA	AA
150	Modok Gier	AA(W)	1.25	2x	++			AA	AA
151	Microcarpa	AA(W)	1.26	2x	++			AA	AA
152	Malaccensis	AA(W)	1.23	2x	++			AA	AA
153	Krasan Saichon	AA(W)	1.22	2x	++			AA	AA
154	Khae	AA(W)	1.28	2x	++			AA	AA
155	Jambi	AA(W)	1.27	2x	++			AA	AA
156	Cici	AA(W)	1.24	2x	++			AA	AA
157	Calcutta 4	AA(W)	1.23	2x	++			AA	AA
158	Burmannica	AA(W)	1.23	2x	++			AA	AA
159	Buintenzorg	AA(W)	1.27	2x	++			AA	AA
160	Birmanie	AA(W)	1.28	2x	++			AA	AA
161	M 61	AA(H)	1.23	2x	++			AA	AA
162	M 53	AA(H)	1.27	2x	++			AA	AA
163	M 48	AA(H)	1.28	2x	++			AA	AA
164	F3P4	AA(H)	1.23	2x	++			AA	AA
165	F2P2	AA(H)	1.25	2x	++			AA	AA
166	Tuugia	AA(C)	1.28	2x	++			AA	AA
167	Tongat	AA(C)	1.23	2x	++			AA	AA
168	Giral	AAB	1.90	3x	++	+	+	AAB	AAB
169	Tjau Lagada	AA(C)	1.23	2x	++			AA	AA
170	Thong Dok Mak	AA(C)	1.24	2x	++			AA	AA
171	TA	AA(C)	1.24	2x	++			AA	AA
172	Sowmuk	AA(C)	1.26	2x	++			AA	AA
173	SA	AA(C)	1.23	2x	++			AA	AA
174	S/N. 2	AA(C)	1.25	2x	++			AA	AA
175	Raja Uter	AA(C)	1.25	2x	++			AA	AA
176	Pipit	AA(C)	1.28	2x	++			AA	AA
177	Ouro	AA(C)	1.23	2x	++			AA	AA
178	Niyarma Yik	AA(C)	1.28	2x	++			AA	AA
179	NBF 9	AA(C)	1.27	2x	++			AA	AA
180	NBA 14	AA(C)	1.23	2x	++			AA	AA
181	Mangana	AA(C)	1.23	2x	++			AA	AA
182	Mambee Thu	AA(C)	1.27	2x	++			AA	AA
183	Malbut	AA(C)	M	?	++			AA?	AA
184	Lidi	AA(C)	1.27	2x	++			AA	AA
185	Khi Maeo	AA(C)	1.27	2x	++			AA	AA
186	Khai Nai On	AA(C)	1.27	2x	++			AA	AA
187	Khai	AA(C)	1.23	2x	++			AA	AA
188	Jari Buaya	AA(C)	1.25	2x	++			AA	AA
189	Jaran	AA(C)	1.26	2x	++			AA	AA
190	Fako Fako	AA(C)	1.28	2x	++			AA	AA
191	Berlin	AA(C)	1.23	2x	++			AA	AA
192	Babi Yadefana	AA(C)	1.26	2x	++			AA	AA
193	Prata Manteiga	AAB	1.92	3x	++			AAB	AAB
194	Borneo	AA (W)	1.25	2x	++			AA	AA
195	Madu	AA	1.28	2x	++	++	+	AB?	AA
196	Prata Maçã	AAAB	2.46	4x	++	+	+	AAAB	AAAB
197	Verde	AAB	1.90	3x	++	+	+	AAB	AAB
198	Prata Anã 2	AAB	1.90	3x	++	+	+	AAB	AAB
199	Prata Anã 3	AAB	1.91	3x	++	+	+	AAB	AAB
200	Pacovan Ken-?	AAAB	2.46	4x	++	+	+	AAAB	AAAB
201	Pitogo	ABB	1.24	2x	++	++	++	AB?	ABB
202	Pacha Nadan	AB	1.97	3x	++	+	+	AAB	AAB
203	Njok Kon	AAB	1.94	3x	++	++	++	ABB	ABB
204	Marmelo	^y^NI	1.25	2x	++	++	++	AB?	ABB
205	Lareina BT100	^y^NI	1.30	2x	++			AA	AAA/AAAA
206	Pisang Ceylan	AAB	?	?	++	+	+	AAB	AAB
207	Pisang Nangka	AA	1.28	2x	++			AA	AAB
208	Willians	AAA	1.92	3x	++			AAA	AAA
209	PV42-114	AAAB	2.28	4x	++			AAAB	AAAB
210	PV03-76	AAAB	2.29	4x	++			AAAB	AAAB
211	Khae Prae	AA	1.23	2x	++			AA	AA
212	Pitu	AA	1.23	2x	++			AA	AA
213	Paka IV	AA	1.26	2x	++			AA	AA
214	Ido 110	AA	1.28	2x	++			AA	AA
215	P.Kermain	NI	1.23	2x	++			AA	AA
216	P.Serum	AA	1.24	2x	++			AA	AA
217	Pisang Mas	AA	1.25	2x	++			AA	AA
218	Uw Ati	AA	?	?	++			AA?	AA
219	Diplóide Bélgica	AA	1.24	2x		++	++	BB	BB
220	BB França	BB	1.27	2x		++	++	BB	BB
221	BB IAC	BB	1.28	2x		++	++	BB	BB
222	*Musa* laterita	*Musa*	1.28	2x	++			-	-
223	Tambi	AAA	1.92	3x	++			AAA	AAA
224	*Musa* royal	*Musa*	1.27	2x	++	++		-	-
Minimal CV (%)			1.23						
Maximum CV (%)			4.56						
Mean CV (%)			3.31						

++: fragment with strong signal; +: fragment with weak signal.

^z^Information based on the *Musa* MGIS database [42].

^y^NI: No information.

^x^M: Mixoploidy; C: cultivated; W: wild; H: hybrid.

^w^ ?: undefined name or ploidy, or unresolved genomic constitution.

^v^Genome composition based on groups formed based on clustering analysis derived from SSR data (Figure 3).

### Flow cytometry analyses

To determine the ploidy, approximately 20 to 30 mg of fresh young healthy leaf tissue from each sample, in addition to the same amount of internal standard *Pisum sativum*[43], were finely chopped with a blade in a Petri dish containing appropriate buffer [44] to lyse the cells and release the nuclei into the suspension. The nuclei suspension was then filtered through a 50 μm screen and stained with 25 μL of 1 mg mL^-1^ propidium iodide, followed by the addition of 5 μL of RNase solution (100 μg mL^-1^). Each accession was represented by samples from three individual with one leaf each. For each sample, at least 10,000 nuclei were analyzed using a FACSCalibur flow cytometer (Becton Dickinson & Co.; San Jose, CA, USA), and histograms with the nuclei counts and fluorescence values were obtained using the software CellQuest (Becton Dickinson). Statistics for DNA content were estimated using WinMDI 2.8 (http://facs.scripps.edu/software.html). The DNA content was expressed in pg (2C), estimated based on the *P. sativum* standard as 2C = 9.09 pg.

### Amplification of the internal transcribed spacers (ITS) for PCR-RFLP

The ITS1-5.8S-ITS2 regions of the nuclear ribosomal gene were amplified using the primers *ITS1* and *ITS4*[45] for the PCR-RFLP method [20]. The amplification reaction (with a final volume of 25 μL and 25 ng genomic DNA) and cycling conditions were identical as proposed by [20], except for primer concentration (0.2 μM of each primer). Five μL of each reaction were used to confirm the amplification by gel electrophoresis. The remaining 20 μL were then digested with 2 U *Rsa*I (Fermentas), after adding 2.5 μL 1X Tango buffer, for 3 h at 37ºC and visualized by 2% agarose gel electrophoresis in 0.5X TBE (90 mM Tris; 90 mM boric acid; 2.5 mM EDTA, pH 8.3) ran for 2 h at 4 V cm^-1^.

**Figure 1:**
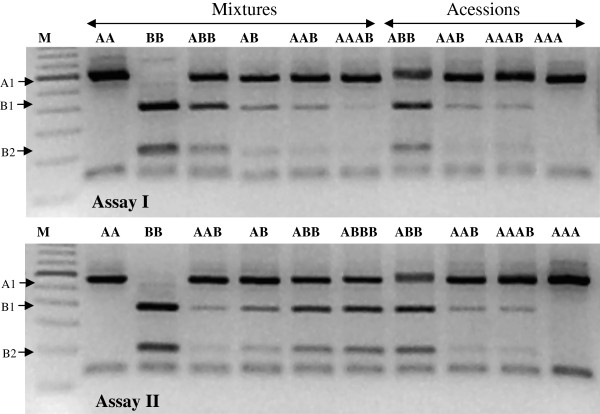
**Restriction profiles of the amplified ITS regions (negative picture).** Assays to verify competition between doses of the A and/or B genomes for amplification and digestion of a rDNA region in *Musa*. Assay I: AA (1AA:0BB); BB (0AA:1BB); ABB (1AA:2BB); AB (1AA:1BB); AAB (2AA:1BB); AAAB (3AA:1BB); ABB, AAB; AAAB; AA. Assay II: AA (1AA:0BB); BB (0AA:1BB); AAB (2AA:1BB); AB (1AA:1BB); ABB (1AA:2BB); ABBB (1AA:3BB); ABB; AAB; AAAB; AA. M: 100 bp ladder.

To discriminate mixtures of genomes at various dosages, the profiles of fragments and band intensities were initially established by sequential mixtures of DNA samples from the *M. acuminata* (AA; ‘Calcutta 4’) and the *M. balbisiana* (BB; ‘Butuhan’) genomes to obtain various artificial combinations of genomes. In a first assay, equimolar amounts of DNA from AA and BB were combined in the following molar proportions: 1AA:2BB; 1AA:1BB; 2AA:1BB; and 3AA:1BB to simulate ABB, AB, AAB, and AAAB, respectively. For the second assay, the ratios 2AA:1BB; 1AA:1BB; 1AA:2BB; and 1AA:3BB were prepared to simulate AAB, AB, ABB and ABBB genotypes, respectively. Accessions 20 (ABB); 53 (AAB); 84 (AAAB); and 142 (AA) with known genomic constitutions (Additional file 1: Table S1) were used as positive controls for both assays (Figure 1).

### Analyses of SSR loci

A total of 21 SSR loci were tested (Additional file 1: Table S2), including two loci from the ‘Ma’ series [46]; three from the ‘AGMI’ series [47]; four ‘Mb’ locus derived from *M. balbisiana*[48]; eight derived from the *M. acuminata* commercial cultivar ‘Ouro’(AA) (*MaO*) [23]; and four new loci, being two from ‘Ouro’ (*MaO-CEN*) and another two from *M. acuminata* ‘Calcutta 4’ (*MaC-CEN*). The amplification reactions contained 25 ng of DNA; 1.5 mM MgCl_2_; 100 μM of each dNTPs; 0.2 μM of each primer and 1.2 U *Taq* polymerase in 1x PCR buffer (Fermentas) in a final volume of 10 μL. The amplifications were conducted using a touchdown cycle [23]. The loci were analyzed in an automatic DNA analyzer, and the amplification reactions were conducted for each locus separately, each with a forward primer containing one of the three additional tail sequences [49] equivalent to a fluorescent primer that was at a concentration of 0.02 μM. An aliquot of 1 μL of each amplification reaction for each one of the three fluorescence of each individual was mixed with 12 μL of Hi-Di formamide (Applied Biosystems) and 0.5 μL of the ROX-500 size standard (35–500 bp) (Applied Biosystems) at an original concentration of 8 nM. This mixture was then denatured at 94ºC for 5 min and kept on ice before injection. The samples were loaded into an ABI PRISM 310 Genetic Analyzer, and the results were analyzed using a GeneScan and Genotyper (Applied Biosystems).

### Statistical analysis of the SSR data

For all accessions (2*x*; 3*x*; and 4*x*), the polymorphic information content (PIC) was estimated for each SSR locus as *PIC*_*i*_ *= 2f*_*i*_*(1 – f*_*i*_*)*, where *i* is the information of the *i*^*th*^ marker; *f*_*i*_ is the frequency of the amplified allele (presence of a band) and (*1 – f*_*i*_) is the frequency of null alleles [50]. PIC was presented as the mean over the various loci. The Marker Index (MI) was estimated as *MI = PIC* x *EMR*, where *EMR* is the effective multiplex relation given by the product between the total number of fragments (*Na*) and the fraction of polymorphic bands (*β =* number of polymorphic bands/total number of bands) [51]. To compare diploids, the PIC and mean heterozygosity (Ho) were estimated using PowerMarker v3.25 [52].

Two approaches were adopted to investigate the genetic structure and diversity among the accessions. In the first case, polymorphisms were treated as binary data (presence or absence). The binary data were then used to obtain a dissimilarity matrix using the Jaccard index employing the software Genes [53]. The matrix was used to run a cluster analysis based on Neighbor-joining [54] using Mega 4.0 [55]. To determine the genetic structure among accessions, a second approach based on the co-dominant nature of the marker was adopted using the Bayesian method implemented using the software Structure 2.3.2, assuming that some fraction of the genome of each individual came from k populations, characterized by their allelic frequencies [31,56]. The input file was prepared accordingly to multiple ploidies [32] with adaptations. As the tetraploid accessions revealed a similar pattern of alleles as triploids, with the majority of the loci displaying from 1 to 3 alleles, all accessions were standardized as triploid. For diploid accessions with more than two alleles and triploids with more than three alleles, the locus with excess alleles was removed from the analysis and considered missing. For the triploid and tetraploid accessions revealing only two alleles, it was necessary to consider one allele as duplicated. Two alternative matrices were generated: one considering the smallest allele in terms of base pairs as duplicated, and the other, based on the largest allele, as duplicated in the matrix. In this way, a triploid with the allelic profile A_1_A_2_ (A_1_ < A_2_) was considered either as A_1_A_1_A_2_ or A_1_A_2_A_2_, creating two files for analysis (Analysis I and Analysis II, respectively). After determining the number of populations (k), the memberships (matrices *q*) of Analysis I and Analysis II according to Structure were compared by Pearson correlation as proposed by Jing et al. [57]. Thus, a high correlation value between matrices would suggest a similar genetic structure among the approaches.

The origin of the modern banana cultivars involved intra- and interspecific hybridizations, and the mixture model and allelic frequency correlated was adopted. A burn-in of 150,000, followed by 70,000 Monte Carlo Markov Chain, was used for each k, varying from 2 to 30, with ten runs for each k. The choice of the likely number of populations was performed based on the highest log value of the likelihood (LnP(K)) and using the method developed by Evanno et al. [58].

## Results

### Ploidy determination by flow cytometry

Leaf samples from each accession were analyzed by flow cytometry to determine ploidy, and the 2C values were estimated in pg (Table 1). The 77 diploid accessions (AA; BB; and *Rhodochlamys*) presented an average of 2C = 2*x* = 1.26 pg, ranging from 1.22 to 1.30 pg. The 115 triploids (AAA; AAB; or ABB) displayed an average of 2C = 3*x* = 1.93 pg, varying from 1.86 to 1.99 pg, whereas the 23 tetraploid accessions (AAAA or AAAB) had a mean of 2C = 4*x* = 2.45 pg, ranging from 2.28 to 2.56 pg (Table 1). The overall average *M. acuminata* genome (A) and *M. balbisiana* (B) was estimated to be 2C = 1.25 pg. The overall coefficient of variation between samples was 3.31%, ranging from 1.23 to 4.56%.

From the 224 accessions evaluated, 221 were from section *Musa* and three were from section *Rhodochlamys.* From the *Musa* section (Table 1; Additional file 1: Table S1), three accessions (204, 205 and 215) had their ploidy defined for the first time, while for another five (54, 80, 123, 201 and 202), the ploidy level was not in agreement with the passport information. For four accessions (56, 102, 206 and 218), it was not possible to determine the ploidy by flow cytometry, whereas five accessions (10, 11, 21, 117 and 183) exhibited mixoploidy (Table 1).

**Figure 2:**
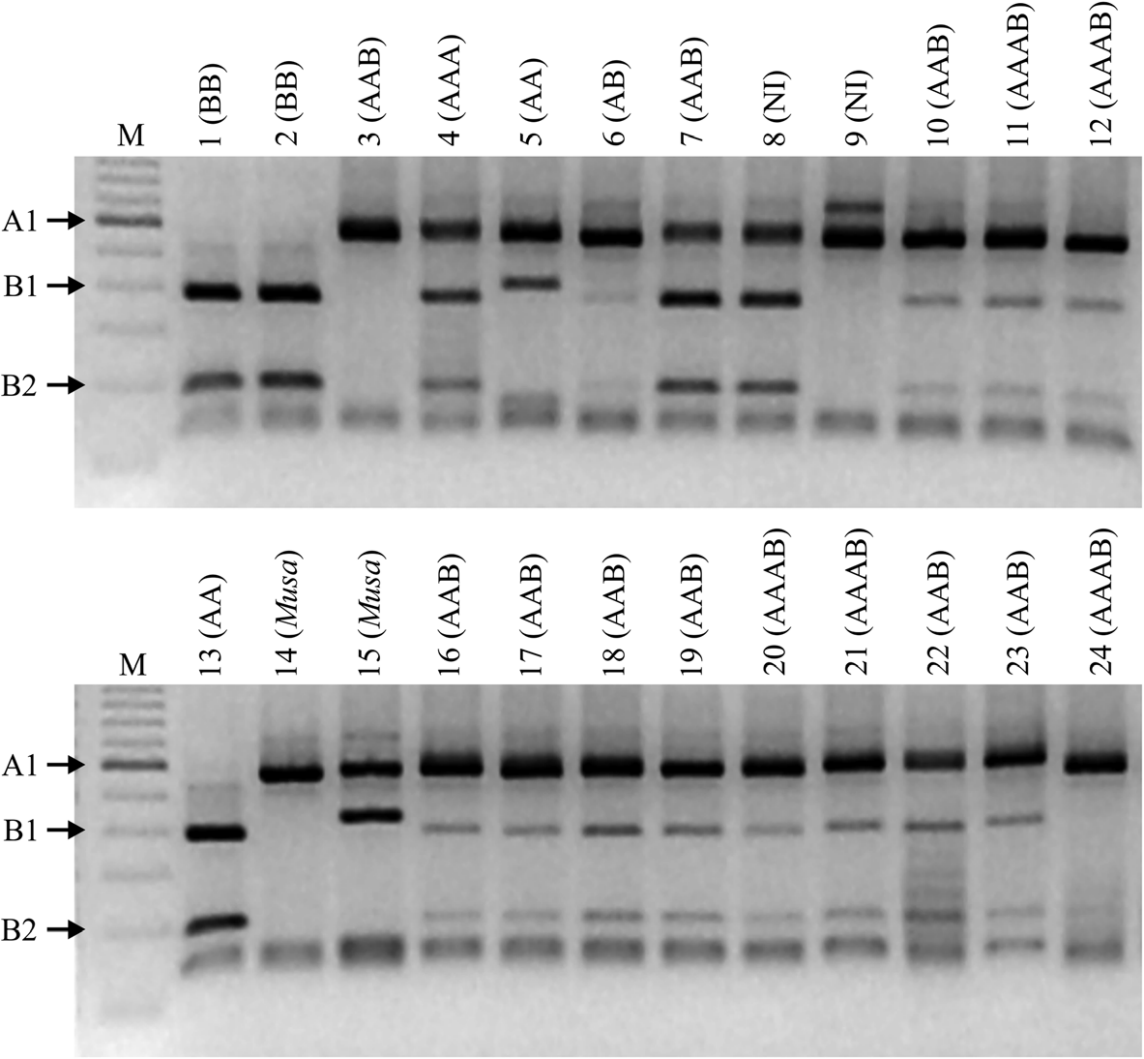
**Restriction profile of the amplified ITS regions from *Musa *accessions with distinct genomic composition.** Amplification products of the ITS1-5.8S-ITS2 region after digestion with *Rsa*I. Accessions 1: ‘Butuhan’; 2: BB‘Panamá’; 3: ‘Figue Rose Naine’; 4: ‘Tugoomomboo’; 5: ‘Madu’; 6: ‘PachaNadan’; 7: ‘Njok Kon’; 8:‘Marmelo’; 9: ‘Lareina BT100’; 10: ‘PisangCeylan’; 11:‘PV42-114’; 12:‘PV03-76’; 13: ‘Diplóide Bélgica’; 14:*Musa laterita*; 15: ‘Musa Royal’ (*M. ornata x M. velutina*); 16:‘Prata Ponta Aparada’; 17:‘Chifre Vaca’; 18: ‘Pulut’; 19: ‘Pratão’; 20:‘Pacovan Ken’; 21:‘Garantida’; 22: ‘Kelat’; 23:‘Java IAC’; 24: ‘BRS Tropical’. Genomic composition determined by morphology is between parentheses. NI no information on genome composition; (*Musa*): accession from *Rhodochlamys*; M: 100 bp ladder marker. Arrows point to fragments of 530 bp specific for A genome (A1); 350 and 180 bp specific for the B genome (B1 and B2).

**Figure 3:**
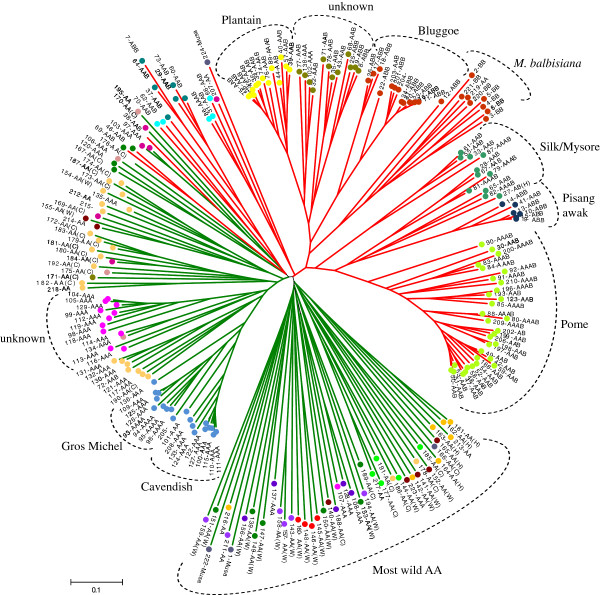
**Phenogram demonstrating the genetic relationships among 224 accessions from the *ex-situ *conservation collection of ‘Embrapa Mandioca Fruticultura’ Center based on 16 SSR loci, obtained using Neighbor-joining clustering from Jaccard dissimilarity index.** Genomic composition based on passport data was included. Full circle colors are related to Figure 6. Accessions containing A genome from *M. acuminata* are shown in green branch line and with B genome from *M. balbisina* in red branch line.

**Figure 4:**
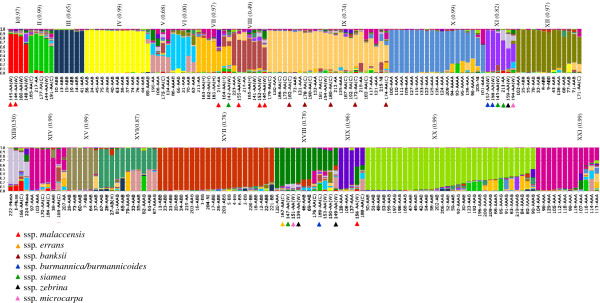
**Diversity structure of the 224 *Musa *accessions based on 16 SSR loci generated by Structure program using the admixture model from matrix derived from Analysis I.** The 21 groups (sub-populations) are represented by distinct colors. Each column represents one accession that can be fractionated into segments, whose size is proportional to the estimated membershipfractions (*q*) in k clusters. Genomic constitutions were based on morphological descriptors (Table 1; Additional file 1: Table S1). Correlation values (r) with the alternative Analysis II used are shown in parenthesis. C: cultivated; W: wild; H: hybrid.

Curiously, accessions 201 (‘Pitogo’) and 204 (‘Marmelo’), classified as diploid by flow cytometry, presented a typical ABB profile by ITS PCR-RFLP (compare lanes 7 and 8, top panel Figure 2). Both accessions were grouped as ABB in the clustering and Structure analyses (Figure 3 and 4 below).

### Characterization of the genomic constitution based on ITS-PCR-RFLP

To evaluate whether the method proposed by Nwakanma et al. [20] would enable the discrimination of genomic constitution and ploidy, preliminary assays were carried out using mixtures of DNA samples from the *M. acuminata* (‘Calcutta 4’) and *M. balbisiana* (‘Butuhan’) genomes to obtain various artificial combinations of genomes, mimicking the natural ones. In the first assay, an increase in genome dose revealed more intense B-specific bands (350 and 180 bp) for BB, followed by ABB, AB, AAB and AAAB (Figure 1; Assay I). A clear distinction between genome composition was possible for BB, ABB and AB, but not between AAB and AAAB. Similarly, no clear difference between the reference genomes ‘Prata Anã’ (53; AAB) and ‘BRS Platina’ (84; AAAB) was detected (Figure 1). In the second assay, the increasing dose of the B genome did not allow the discrimination between ABB and ABBB (Figure 1; Assay II), but both differed from AAB and AB in the band intensity pattern. Thus, this simulation demonstrated the possibility of genome constitution discrimination for accessions when the ploidy level had been previously determined.

Amplification of the ITS regions produced a fragment of ~ 700 bp from all 224 accessions and disclosed the expected fragments that characterized the presence of genome A and/or B after digestion with *Rsa*I (Figure 2). From the 224 accessions evaluated, three accessions without previous information (204, 205 and 215) had their genomic constitution defined, while 13 (5.8%) disagreed with the information available about genomic constitution defined based on previous published or characterized by morphological descriptors, including accessions 7, 10, 11, 28, 68, 72, 79, 102, 195, 201, 202, 203, and 219 (Table 1). But from these 13 accessions, only four (28, 79, 102 and 195) appeared to truly demonstrate inconsistencies for the genomic constitutions established by PCR-RFLP. Accessions 28 (‘Yangambi n^o.^2’; AAB) and 79 (‘BRS Tropical’; AAAB) did not exhibit the B-specific 350 bp fragment upon digestion, while 102 (‘Tugoomomboo’; AAA) displayed a typical ABB digestion pattern, and accession 195 (‘Madu’; AA) presented a slight deviation in size of the B-specific fragment. By clustering analysis derived from SSR genotyping (see below), genomic constitution for accessions 28, 79, 102 and 195 were confirmed as AAB, AAAB, AAB, and AA, respectively.

For the *Musa* ornamental diploid species represented by *M. basjoo* (accession 1; Table 1) and the hybrid ‘Royal’ (224), derived from a cross between two species of the section *Rhodochlamys* (*M. ornata* x *M. velutina*) [59], a slightly larger fragment than the 350 bp from *M. balbisiana* and the 530 bp from the *M. acuminata* fragment were observed. For *M. laterita* (222; section *Rhodochlamys*), only the typical *M. acuminata* 530 bp fragment was detected (Figure 2; Table 1).

### SSR and genetic diversity analyses

Of the 21 loci tested, only five (*MaOCEN09*; *Mb1-69*; *Mb1-134*; *Mb1-139*; and *AGMI24-25*) failed to amplify consistently, while sixteen SSR loci successfully amplified 182 alleles from the 224 accessions, with an average of 11.5 alleles per locus and a range from 7 to 15 alleles (Additional file 1: Table S2). The discriminatory power of each locus was evaluated by estimating the Polymorphic Information Content (PIC) and the Marker Index (MI). To estimate the PIC, the microsatellite data were converted into a binary format (presence or absence of bands), and therefore, the maximum PIC could be 0.5. The average PIC over 16 loci was 0.20, ranging from 0.16 to 0.30 per locus, indicating a large discriminatory power for the analyzed loci (Additional file 1: Table S2). The MI [51,60] ranged from 1.57 for *MaOCEN03* to 3.24 for *MaC-CEN04*, with an average of 2.28. Considering the mean value of 2.28 as a reference, seven loci (*Ma1-17*; *AGMI 93/94*; *MaOCEN01*; *MaOCEN10*; *MaOCEN14*; *MaOCEN19*; and *MaC-CEN04*) revealed more diversity in the banana (Additional file 1: Table S2).

Overall, regardless of ploidy, there was a predominance of accessions with two alleles (35.2 to 55.8%), followed by those with one (14.1% to 60.7%); three (3.5 to 32.8%); and only a small fraction with four alleles (0.3 to 15.6%) (Table 2). BB and ABB were the groups with the largest proportion of accessions displaying a single allele (60.7% and 41.9%, respectively), followed by wild (41.3%) and cultivated AA diploids (39.7%). A small fraction of diploid accessions revealed three alleles in cultivated AA (4.2%), BB (4.1%) and wild AA (3.5%). Accessions with three alleles predominated in triploids (ranging from 18.3% for AAB to 24.3% for AAA) and tetraploids (28.2% for AAAB and 32.8% for AAAA). Few accessions revealed four alleles, mostly were tetraploid hybrids AAAA, with 15.6% of accessions, and AAAB with 3.0% (Table 2).

**Table 2:** Average ratio (in %) of accessions per genomic groups, presenting one, two, three or four alleles

**No.alleles**	**Genomic group**^**Z**^							
	**BB**	**AA (W)**	**AA (C)**	**AAA**	**AAB**	**ABB**	**AAAB**	**AAAA**
	**(%)**							
1	60.7	41.3	39.7	22.7	31.7	41.9	21.8	14.1
2	35.2	54.3	55.8	52.6	48.7	39.4	47.0	37.5
3	4.1	3.5	4.2	24.3	18.3	18.4	28.2	32.8
4	0.0	0.9	0.3	0.5	1.3	0.3	3.0	15.6
**n**	8	23	26	43	56	21	16	4
**n**_**m**_	7.6	21.6	24.1	42.8	51.0	20	14.6	4

^Z^W: Wild accessions; C: Cultivated; n: number of samples evaluated; and n_m_: mean number of samples for the evaluated loci.

**Figure 5:**
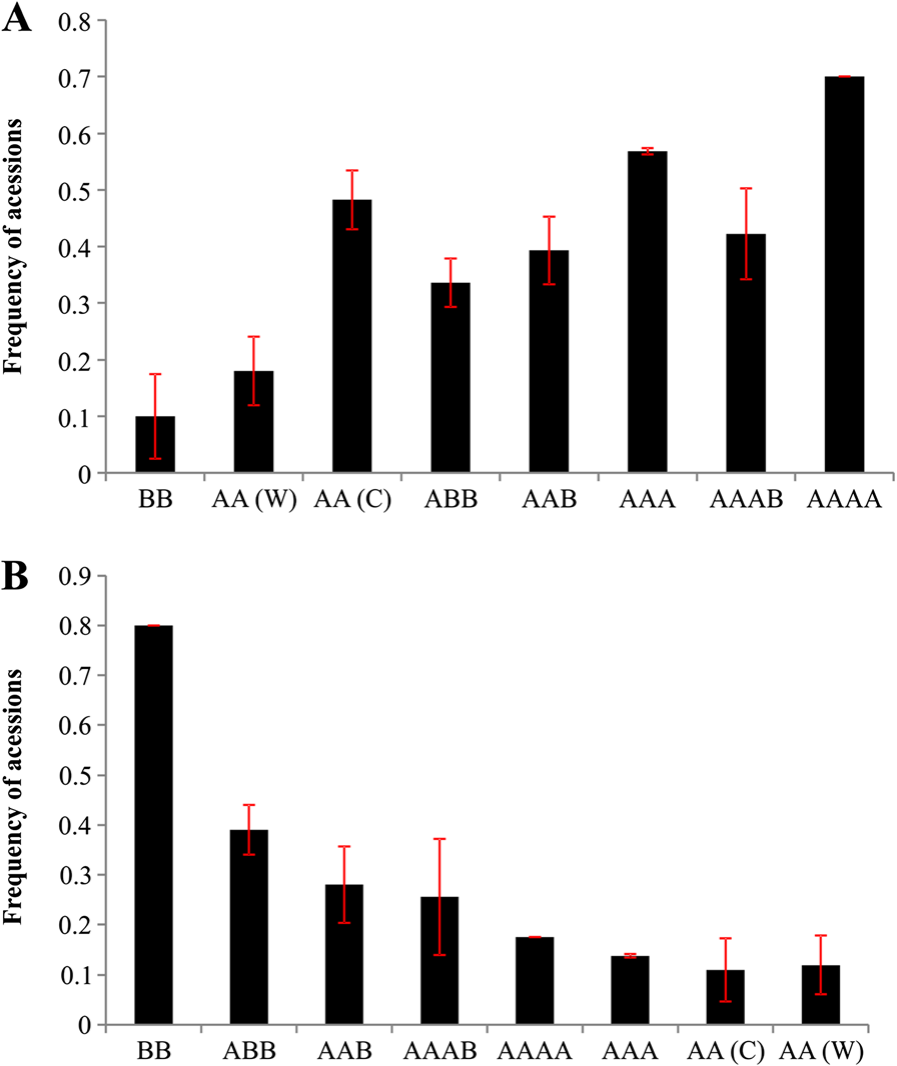
**Frequency distribution of the 20 most frequent alleles in cultivated diploid accessions AA(C) (panel A) and BB (panel B) in comparison with other genomic groups.** W: wild; C: cultivated. The errors bars refer to the ratio of accessions that did not amplify one or more analyzed loci.

The relationship among the 20 most frequent alleles in the cultivated AA and BB accessions was investigated in relation to the other genomic and ploidy groups. In general, the most frequent alleles in cultivated AA tended to increase in frequency according to the dose of the A genome (*M. acuminata*) in the higher ploidy genomic groups (Figure 5A). Similarly, the most frequent alleles in BB decreased proportionally with the reduction in the dose of the B genome (*M. balbisiana*) in the accessions (Figure 5B).

Cultivated diploids displayed higher mean heterozygosity (62.4%) than the wild diploids (overall average 56.4%). The lowest mean heterozygosity (37.4%) was detected among the *M. balbisiana* accessions (Additional file 1: Figure S1), while *M. acuminata* ssp. *microcarpa* and *M. acuminata* ssp. *burmannica/burmannicoides* revealed the largest mean heterozygosity (74% and 71.9%, respectively). The lowest PIC values were detected for the BB accessions and *M. acuminata* ssp. *banksii* with 34.2% and 36.6%, respectively.

### Clustering analyses of the collection

Clustering analysis based on Neighbor-joining essentially allowed the detection of two major clusters (Figure 3). The first cluster contained accessions with at least one copy of the B genome, while the second one contained those exclusively with the A genome (Figure 3), with the exception of the AAB accessions 38, 46, and 69, allocated together with genome A accessions (Table 1). Similar grouping was obtained by Structure analysis (Figure 4). Within these two main clusters, sub-clusters were formed with accessions according to genome composition and ploidy level. Within the major A or AB clusters, the main clusters usually corroborated the classification of subgroups, such as ‘Pome’ and derived hybrids; ‘Plantain’; ‘Silk’; ‘Pisang awak’; ‘Bluggoe’; ‘Cavendish’; and ‘Gros Michel’ (Figure 3). Accessions without previous classification were allocated into the main subgroups, allowing novel categorization, while two sub-clusters (denominated ‘unknown’ in Figure 3) require further investigations to define proper subgroup classification. Some accessions did not differ for their SSR profiles, possibly representing duplicated accessions (Figure 3), including accessions 45 and 63 from the ‘Plantain’ subgroup; 15 and 19 from ‘Pisang awak’ (ABB); 11 and 16, and 20, 21, and 24 from ‘Bluggoe’ (ABB).

### Population structure analysis

**Figure 6:**
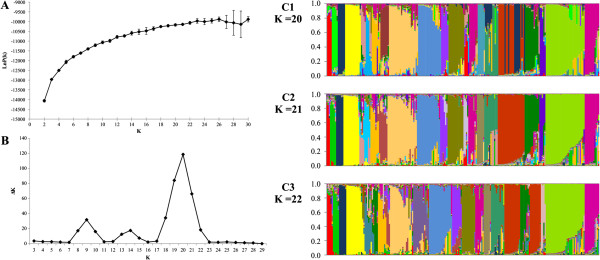
**Left panel: Selection of the most likely number of subpopulations (k) for the evaluated accessions. A**. Mean values of LnP(K) for 10 independent runs for each k. **B**. Plot of Δk values for each k based on the second order change of the likelihood function. Right panel **C**. Graph for ancestralities for k = 20 (C1), k = 21 (C2), and k = 22 (C3). Group colors are function of colors observed for k = 21.

The co-dominant nature of the SSR markers was exploited to analyze the structure of the populations using a Bayesian approach. The number of subpopulations (k) tested ranged between 2 and 30 (Figure 6A). To estimate the approximate number of subpopulations, the maximum estimated value of the logarithm of likelihood (LnP(K)) was used. However, for the evaluated accessions, the value for LnP(K) did not reach a clear plateau, continuing to increase together with the variances between the tested k (Figure 6A). Under these circumstances, the number of subpopulations (k) was projected to be between 16 and 23 (Figure 6A). For k = 20, 21 or 22, there was no large variation for the main groups formed (Figure 6; panels C1, C2 and C3). The method that calculates the second order of likelihood change (Δk) is more sensitive than the previous one to detect the number of subpopulations under these circumstances [58]. Adopting this approach, Δk peaked at k = 21 (Figure 6B).

The two alternative matrices tested (Analysis I and II) presented little differences for genotype allocation and membership values (*q*). The Pearson correlations (*r*) between the two distinct alternative approaches was high and significant (p ≤ 0.01) for most groups (r = 0.65 to 0.99), indicating a good adjustment between the co-ancestries that the alternative matrices generated (not shown), except for group VI, which did not show any correspondence between the two analyses (Figure 4). Therefore, only results from Analysis I (see Methods) was used for the purpose of discussion.

From the 21 groups formed by Structure (Figure 4), five contained only diploid accessions (group I, II, VII, XI, and XIII); six contained triploids or tetraploids (III, IV, VI, XV, XX, and XXI); and the other ten contained mixtures of diploids and triploids with the following (2*x*:3*x*/4*x*) proportion for each group: V (2:2); VIII (6:1); IX (17:7); XII (1:12); XIV (4:3); XVI (1:10); XVII (9:13); XVIII (9:3); and XIX (1:3); and X (1 2*x*: 14 3*x*: 4 4*x*).

**Figure 7:**
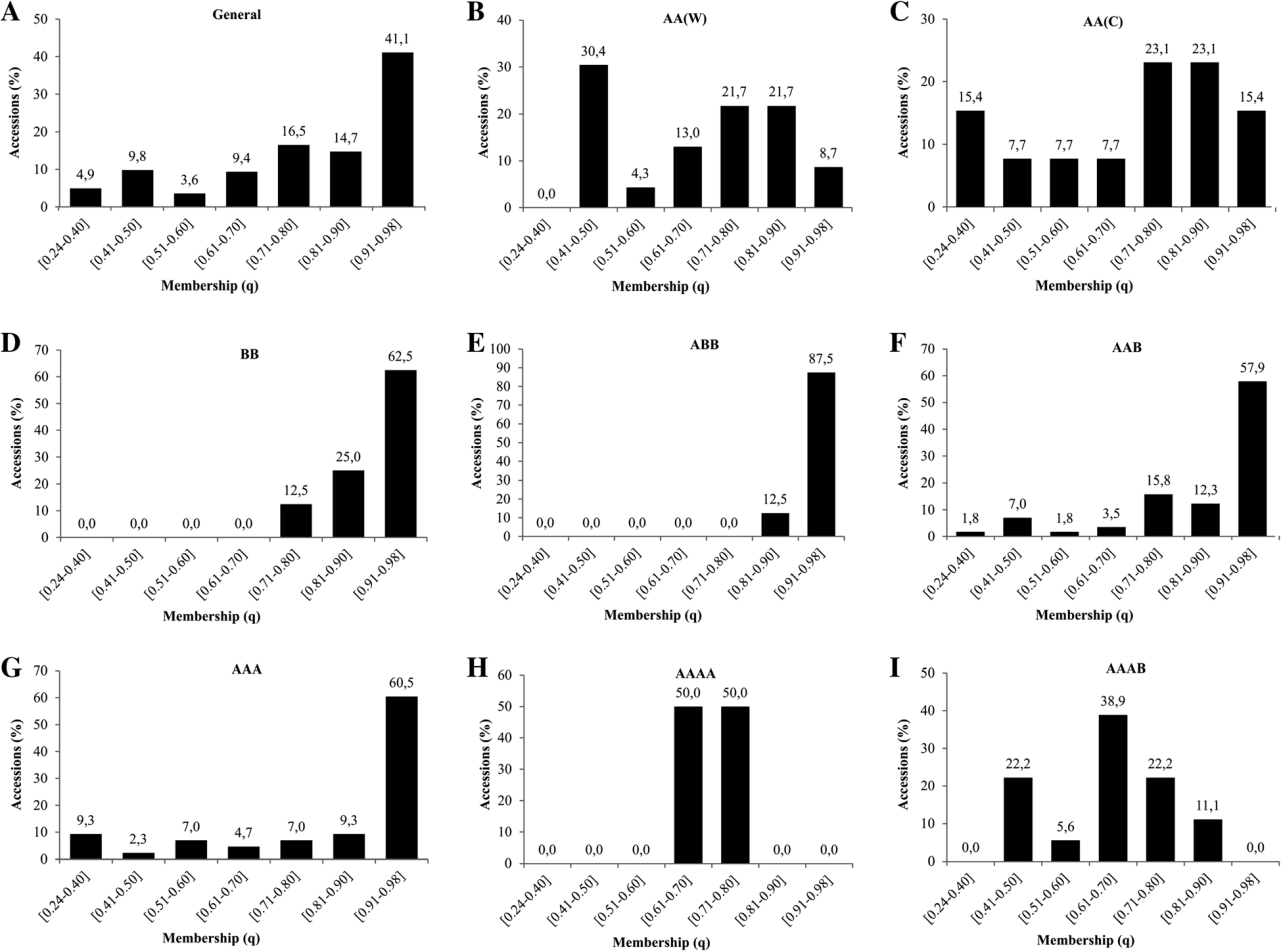
**Percent of accessions within intervals of membership (*q*) for all accessions: A) general; B) wild AA [AA(W)]; C) cultivated AA [AA(C)]; D) BB; E) ABB; F) AAB; G) AAA; H) AAAA; and I) AAAB.**

The membership value (*q*) for the 21 subpopulations (224 accessions) varied from 0.24 to 0.60 for 41 accessions; 0.61 to 0.80 for 58 accessions; 0.81 to 0.90 for 33 accessions; and greater than 0.90 for 92 accessions (Figure 7A). The largest frequencies of accessions with higher membership (0.90 < *q* ≤ 0.98) were from the genomic groups ABB; BB and AAA with 87.5%; 62.5%; and 60.5%, respectively (Figure 7E; D; G). On the other hand, the lowest values of membership (*q* varying from 0.24 to 0.50) were observed for the wild AA diploids [AA(W)], the cultivated diploids [AA(C)], and AAAB, at 30.4%, 23.2%, and 22.2% of accessions, respectively (Figure 7B; C; I). Accessions from the main banana cultivated subgroups (AAA, AAB, ABB) in general exhibited high membership values (Figure 4), but accessions with admixture (*q* ≤ 0.90) were also encountered, such as 43, 71, 68, 77, and 138 in group XII (‘Saba’ subgroup); accessions 28, 33, 55, 61, 65, and 67 in group XVI (‘Silk’/‘Mysore’ subgroups); 101, 136 and 208 in group X (‘Cavendish’/‘Gros Michel’ subgroups); accessions 30, 52, 59, 193, and 206 in group XX (‘Pome’ subgroup); and 107, 113, 114, and 116 in group XXI. Other triploid accessions with admixture were distributed in groups V; VI; VII; IX; XII; XIV; XV; XVII; and XIX (Figure 4).

Essentially, the triploid/tetraploid groups generated by Structure were identical to the clusters revealed by clustering analysis for the major banana subgroups, such as ‘Pisang awak’ (group III; Figure 3 and 4); ‘Plantain’ (group IV); ‘Cavendish’ and ‘Gros Michel’ (group X); ‘Bluggoe’ (group XVII); ‘Pome’ (group XX), and groups XV, XII and XXI with non-categorized accessions (Figure 3 and 4).

Regarding the diploid accessions analyzed by Structure, all eight *M. balbisiana* accessions were placed in sub-population XVII, together with 12 ABB accessions (80%) (Figure 4). The *M. acuminata* subspecies (Additional file 1: Table S1) were distributed into various clusters: ssp. *malaccensis* with two accessions at group I; one at VII; three at VIII; and one at XIX; ssp. *errans* with one accession at group XVIII; ssp. *banksii* with 5 accessions at group IX; spp. *burmannica/burmannicoides* with two accessions at XI; and one at XVIII; ssp. *siamea* with one accession at VII; two at XI; and one at XVIII; ssp. *zebrina* with one accession at XI; and two at XVIII; and ssp. *microcarpa* with one accession at XI; and two at XVII (Figure 4).

Diploid accessions were highly heterogeneous (mixture), and their ancestry remained restricted to other group of diploids, except for accessions 161, 162, 183 and 195, which exhibited ancestry with group XXI of AAA triploids, and BB ‘IAC’ (221) with ancestry to group III of the subgroup ‘Pisang awak’ (ABB) (Figure 4).

## Discussion

### Characterization of ploidy and genomic constitution

Flow cytometry was used to define the genome size (2C content) and the ploidy level of 224 accessions. From the 221 section *Musa* accessions, only five (2.3%) presented conflicting results with the passport data. Similar discrepancy between estimation of ploidy by morphological characterization and flow cytometry had been reported [61,62]. Previously, it was believed that nuclear DNA content would be a good predictor of genomic constitution [63], as the BB genome was thought to be on average 12% smaller than the AA genome [64]. However, in our study the estimated size of genome A or B did not differ among the various ploidies and genomic groups, and therefore, estimating C values by flow cytometry alone could not distinguish the genomic constitution. The predicting value of genomic constitution might be affected by minute differences in the size of individual A and B genomes; variation in the number of sets of chromosomes from distinct genomes in triploids or tetraploids, including the occurrence of aneuploids [65]; the involvement of other *Musa* genomes, such as the presence of S or T genomes (from *M. schizocarpa* or *M. textilis*, respectively) in some cultivars [65]; or the lack of additiveness of genome size, caused by recombination, resulting in different proportions of genomes A or B [66,67].

Determination of genomic constitution by molecular markers has long been sought, with attempts to use RAPD [68] or SSR [23,28,47,69,70], but with limited precision to determine the genome dosage. When we evaluated the ITS PCR-RFLP approach using standard cultivars, it was possible to identify all expected digested fragments, except the smallest one (50 bp) reported by Nwakanma et al. [20], which was not predicted by *in silico* digestion (not shown). Simulating the various A and B genome constitution and dosages indicated the ability to distinguish most genome combinations (BB, AAB, ABB and AB); however AAB could not be distinguished from AAAB, and ABB could not be distinguished from ABBB, possibly because of amplification competition. For successful adoption of this approach, knowledge about ploidy is essential [20]. When the ITS PCR-RFLP approach was applied to the whole collection, the genomic constitution of most of the accessions was congruent with the morphologic classification available, as previously reported [21]. Our data indicated that determination of ploidy and genomic constitution using morphologic descriptors can still be considered reliable and useful in most cases, with few exceptions.

Noteworthy, our study revealed that a few accessions presented unexpected behavior, such as ‘Yangambi no.2’ (28) and ‘BRS Tropical’ (79), recognized as AAB and AAAB, respectively, but they exhibited typical AAA and AAAA digestion profiles. These changes in the restriction profiles for ‘Yangambi nº 2’ and ‘BRS Tropical’ (a tetraploid hybrid from ‘Yangambi nº 2’) might have derived from a variant of the B genome rDNA-locus. Other unusual alleles were identified. For example, ‘Tugoomomboo’ (102), considered as AAA, exhibited an ABB PCR-RFLP profile, but it was classified as AAB by clustering analysis, suggesting the occurrence of the B genome allele for the ITS regions in one of the A genomes. The diploid AA ‘Madu’ (195) was indicated to be AB, with a slight change in the restriction fragment size for the B genome. This alteration in size was derived from a change in the *Rsa*I restriction site, later confirmed by sequencing (not shown). This accession also exhibited ancestrality from group VI of AAB and AAAB and XVIII of AAA/AA/AAB (Figure 4). Such results can be related to the occurrence of recombination between the A and B genomes [5,66,67].

Incomplete concerted evolution of ITS sequences observed in *Musa* hybrids, with the predominance of the original parental alleles, might derive from the absence of sexual reproduction [71]. But the observation of unexpected genotypes, demonstrated by sequence analyses of ITS and ETS regions of rDNA, have pointed to the occurrence of recombination between A and B or between *M. acuminata* subspecies genomes [5,20,71]. Homeologue pairing and recombination between A and B chromosomes have been actually observed in meiosis of triploid hybrid accessions (AAB and ABB) and an allotetraploid (AABB), and appeared to occur at some frequency [66,70].

Therefore, despite fact that small differences in genome size between *M. acuminata* and *M. balbisiana* are recognized, the occurrence of chromosome recombination and multivalent pairing during meiosis, leading to unbalance genome segregation, could generate a continuum in genome sizes among accessions, overlapping differences and impairing the ability to distinguish genomic constitution, as corroborated by our results and others [61,62]. Similarly, our results from PCR RFLP of ITS sequences pointed to the occurrence of recombinants, with the lack of B alleles in two hybrid accessions (AAB and AAAB), or the B genome allele in one of the A genomes for a ABB and AA. Exceptions from the commonly observed incomplete concerted evolution might be associated with the occurrence of sexual reproduction, with meiosis offering the possibility for homeologue chromosome pairing generating recombinant chromosomes.

### Genetic diversity and clustering analysis

Sixteen SSR loci were used, revealing 182 alleles, with an average of 11.5, while Christelová et al. [29] detected an average of 15.4 and 14 alleles for 70 diploid and 38 triploid accessions, respectively. Within each ploidy level, the BB genome group presented a higher proportion of accessions with only one allele (homozygosis) as previously reported [7], suggesting a lower genetic variability [72] or the occurrence of a large number of null alleles among the accessions evaluated. Conversely, in cultivated AA accessions, structural heterozygosity [9,73] might justify larger average heterozygosity (62.4%), as well as limited fertility [7,9,73,74], in comparison to the wild diploids (mean 56.4%) (Additional file 1: Figure S1). Previous studies reported heterozygosity of 61% for cultivated AA and 53% for wild diploid accessions based on SSR markers [26], and 61% for cultivated AA and 53% for wild AA using RFLP markers [7].

In our study, it was verified a high proportion (more than 75%) of accessions producing one and two alleles among triploids. Banana triploid cultivars supposedly originated from crosses between non-reduced 2*n* gamete (restitution of the first or the second division) and reduced *n* gamete. The formation of non-reduced gametes tends to be higher when two different genomes are involved, such as in the case of AB or AA hybrids between subspecies of *M. acuminata*, as in the cultivated diploids [8,9]. In the case of triploids, they most likely resulted from crosses between heterozygote diploid individuals, such as the cultivated diploids with non-reduced gametes (2*n*) and another individual (*n*) carrying a similar allele to one found in the other parent. This hypothesis is supported by the finding that the most frequent alleles found in cultivated AA diploids were observed in increasing frequency in triploid and tetraploid accessions, containing increasing dosages of the *M. acuminata* genome (Figure 5A). The association with cultivated diploids is justified by the presence in cultivated triploids and tetraploids of domestication traits, such as parthenocarpy, sterility and pulp yield [9]. Further, Ortiz [75] investigated the occurrence of non-reduced gametes and observed that all genotypes that produced 2*n* gametes also produced fruits by pathernocarpy. Many cultivated triploids presented the same mitochondrial and chloroplast patterns as the cultivated diploids [2]. The *M. acuminata* spp. *banksii* and *M. a.* spp. *errans* subspecies, characterized as cultivated diploids, are involved in the development of almost all the cultivated diploids and triploids and parthenocarpic cultivars [2,9,10].

Despite the fact that there was a trend of the participation of AA(C) in some accessions, only 34% (ABB); 39% (AAB); 57% (AAA); 42% (AAAB); and 70% (AAAA) of the accessions contained such alleles. This fact reinforces the previous observation from PCR-RFLP, that the origin of cultivated bananas might have involved recombination events (inter- and intraspecific) and backcrosses between species as well as human intervention. Therefore, a cultivar cannot carry the whole allelic complement from a specific genome A or B [66]. On the other hand, 40% of the alleles present in the eight BB accessions were not detected on ABB, most likely because there is a larger diversity of BB in the formation of ABB. Hippolyte et al. [76] also verified a larger diversity in the B genome of interspecific hybrids, such as ABB, than in BB, suggesting an under-representation of the *M. balbisinana* diversity or the extinction of the parental donor of the B genome in these hybrids. Our study also detected these differences (Additional file 1: Figure S2), but when compared to BB, ABB showed to be more uniform (*q* > 0.91 for 62.5% and 87.5% of accessions) in the Structure analysis (Figure 7 and 4).

The analysis performed by converting SSR genotyping into binary data and using it to estimate dissimilarities among genotypes revealed a broad genetic variability among *Musa* accessions (Additional file 1: Table S2). SSR loci enabled the separation of accessions into two major clusters (one with at least one copy of the B genome, and the second with those exclusively with the A genome) and according to genomic constitution. Further subdivision, in general, corroborated the classification into banana subgroups (‘Pome’, ‘Plantain’, ‘Cavendish’, ‘Gros Michel’, ‘Bluggoe’ ‘Silk’, and ‘Pisang awak’). The most diverse accessions were AA diploids and the less diverse were subgroups of commercial interest, such as ‘Pome’, ‘Plantain’, ‘Cavendish’, ‘Gros Michel’, and ‘Bluggoe’, corroborating previous studies [21,22,28,29,70,77-79]. Banana subgroups are characterized by genotypes that share similar agronomic and fruit quality traits [22], which are believed to originate from a common ancestor, meaning, one single meiotic event and the total lack of a sexual stage in the evolution of these subgroups [78], which justifies the small genetic differences. However, large morphological differences are observed in the field maintained by asexual propagation [78-80]. Epigenetic regulation might help to elucidate phenotypic differences within subgroups not correlated with genetic differences [66,76].

In addition to the contribution regarding the identification of duplicated accessions, definition of the ploidy level and genomic constitution of the accessions, the cluster analysis based on SSR also enabled us to infer to which subgroup the natural triploid accessions belong, according to their allocation in the phenogram. This is a key aspect because it enabled us to separate accessions with similar agronomic attributes. This information can be used by breeding programs to develop hybrids, which requires certain agronomic or qualitative requisites of the subgroups. However, two clusters (identified as ‘unknown’; Figure 3) need to be further investigated for proper categorization.

### Population structure and genetic relationships of accessions

To our knowledge, this is the first work to explore the co-dominant nature of the SSR markers in *Musa* accessions with distinct ploidy levels using the Bayesian model from Structure. Establishing the relationships and evolution of the genomes of modern cultivars, landraces and their wild relatives is of great importance to determine the effect of human intervention on the process of domestication and to understand the geographic dimension of the diversity and the domestication process of wild species [11]. Many species have undergone a long and complex period of domestication and breeding with limited gene flow, it is expected that there is a complex population structure [81,82].

Here, we suggested the separation of 224 accessions into 21 subpopulations (groups) based on the method proposed by Evanno et al. [58]. Such elevated number of groups was expected considering that accessions with different genomic constitution (AA, BB, ABB, AAB, AAA, AAAA, and AAAB), and from distinct subgroups (‘Pome’, ‘Plantain’, ‘Cavendish’, ‘Gros Michel, etc) from the variou genomic groups were analyzed. In general, the grouping by Structure, even considering some alleles missing, was congruent for most groups formed (triploid and tetraploid accessions, especially) in the phenogram generated based on SSRs as dominant markers (without the exclusion of alleles). The agreement between both sets of data showed that the adaptations did not jeopardize the information from the alleles used in the Structure analysis, which also incorporates ancestrality to each group.

There are emerging evidences that the process of evolution of cultivated bananas might have not derived simply by hybridization followed by selection and clonal propagation (“single-step domestication”), but, on occasions, episodes of meiosis, recombination and fertilization might have eventually occurred [5,66,71]. In our study, evidence of mixed population ancestry, given by membership value (q ≤ 90%) was verified for wild and cultivated diploids, similar to what was observed for tetraploid hybrids from breeding programs. For triploid accessions, there was evidence of admixture (12.5% of ABB accessions; 39.5% of AAA; and 42.1% of AAB) with ancestry mostly in two, or many groups (with minimal ancestry to each group), suggesting multiple origins and/or the occurrence of recombinations more often than expected. However, accessions from subgroups ‘Plantain’ (group V), ‘Cavendish’ and ‘Gros Michel (X), and ‘Pome’ (XX) were highly homogeneous, with a few exceptions.

The subgroup ‘Pome’ (AAB; group XX; Figure 4) contained the most cultivated accessions in Brazil, and the Embrapa´s breding program has focused on the development of tetraploids derived from crosses between a partially fertile cultivated female parent (AAB), producing non-reduced gametes (2*n*), with a male diploid pollen-donor (AA), with novel desirable characters, such as disease resistance. Here, all these ‘Pome’ tetraploid hybrids from Embrapa demonstrated ancestry to the parental diploids ‘M53’ (Group IV) or ‘Calcutta 4’ (Group XI). Similar to what was observed for ‘Pome’ tetraploid hybrids, all the improved AAAA hybrids from ‘Gros Michel’ (94, 95, and 96) presented ancestry to diploid groups VII or II. In the ‘Pome’ subgroup (XX), from five triploids inferred as mixture, only 59 and 193 displayed a clear ancestrality to groups XVI and II, respectively. Curiously, ‘FHIA-02’ (91) is reported to be an AAAA hybrid, from a cross between ‘Williams’ and the diploid ‘SH3393’ with characteristics of the ‘Cavendish’ subgroup [83], but here it presented only 22% of the genome as ‘Cavendish’, suggesting to be ‘Pome’ (Table 1; Figure 3 and 4). Other FHIA hybrids, whose diploid parents were probably not represented in this study displayed ancestry in groups X (‘Cavendish’/‘Gros Michel’), XVI (‘Silk’/‘Mysore’) and XIX (Figure 4).

‘Cavendish’ and ‘Gros Michel’ were separated into two close subgroups in the cluster analysis (Figure 3); however, according to Structure (Figure 4), representative accessions from these subgroups appeared in the same group, most likely because they share common alleles [2,8]. Similar results were also observed using RFLP [8], microsatellite [22], and DArT markers [84], while sharing the same cytotype for organellar genomes as shown based on PCR-RFLP [85]. Hippolyte et al. [76] proposed that accessions from subgroup ‘Cavendish’ and ‘Gros Michel’ are derived from a common 2*n* gamete donor, and most likely two different, but genetically close, *n* donors. Raboin et al. [8] proposed the accessions ‘Sa’ and ‘KhaiNai On’ as the probable *n* gamete donor for ‘Gros Michel’ subgroups. In our study, two diploids with identical denominations (173 and 186) were allocated to group IX, but only accession 136 (‘Amritsagar’) from group X (‘Cavendish’/‘Gros Michel’) presented ancestrality (*q* ~ 18%) to group IX, which gives support to the proposed diploid origins of subgroup ‘Cavendish’ and ‘Gros Michel’. In addition, the diploid ‘Lareina BT100’ (205) was placed in group X and it could be a potential 2*n* gamete donor for ‘Cavendish’ and ‘Gros Michel’. Therefore, diploids from group IX and ‘Lareina BT100’ appeared as potentially related parentals of the ‘Cavendish’ and ‘Gros Michel’, which could be used in crossing programs or chromosome manipulations (doubling) to obtain/re-synthesize ‘Gros Michel’/‘Cavendish’ hybrids.

Noteworthy, some AAB and AAA triploid accessions demonstrated ancestry to other groups, containing other accessions with similar genomic constitution. It is known that some hybrids showed various degree of residual fertility and it is possible that their evolution involved episodes of sexual reproduction, as suggested by the backcross hypothesis [66].

Our results indicated that Structure was efficient in the detection of ancestry of recently developed tetraploid hybrids by breeding programs in Brazil (‘Pome’) and Jamaica (‘Gros Michel’) with a defined genealogy, and for some triploid cultivars. However, this approach appeared to be less efficient to detect the ancestry of most of the primeval triploid accessions, which make up the main commercial subgroups (‘Pisang awak’; ‘Gros Michel’; ‘Cavendish’; ‘Pome’; ‘Plantain’). This absence of detection of ancestry might be explained by a series of hypotheses.

One possibility is that potential parental diploids for the main commercial subgroups were under-represented in the collection, such as demonstrated by the absence of ancestry in diploids groups for some recent tetraploid hybrids developed by FHIA evaluated in this study (Figure 4). Secondly, the long and uncertain evolutionary period that these triploid cultivars went through since they originated might have resulted in changes/mutations in loci, which could result in complete elimination or modification of the alleles in one of the parents. The ability to detect ancestry for recently developed tetraploid hybrids is important evidence supporting this hypothesis. The process of allopolyploidization can lead to activation of retrotransposons; elimination and rearrangements of parental chromosomes [86,87], DNA sequence losses, apparently from the largest parental genome [66,88] and from highly repetitive sequence regions [89]. Such events might have occurred in *M. acuminata*, with a larger genome [62,63] and more repetitive sequences than *M. balbisiana*[90]. Thirdly, the limited number of loci used can also be a reason for the lack of precision in identifying the ancestry of commercial accessions, as a large number of loci would increase the chances of finding equivalent alleles in a group of conserved polymorphic loci among the cultivated triploids and the ancestral diploids. For example, other researchers did not find differences between accessions of the ‘Cavendish’ subgroup [22], but differences between the accessions of this subgroup have been identified here and by Christelová et al. [29], most likely because of the larger number of alleles identified per locus.

The relationship between diploids and AAB could have been affected by the potential occurrence of recombinations between homeologue chromosomes with distinct structural organization, contributiong to large genetic changes in allopolyploids [88]. Recombinations between the A and B genomes can occur, and it can be frequent in triploid hybrids, while it might lead to unbalanced genome transmission with respect to the parental species [66,67], justifying variations in AAB genomes, morphological expression of A and B characters, and no addictiveness, as hybrids may carry different recombinant A and B chomossomes (*e.g.* A^B^ and B^A^) [66]. Therefore, all these processes, occurring in isolation or combined, especially in *M. acuminata* subspecies can obstruct the inference of ancestry for most of the triploid accessions.

Concerning diploids, the groups formed by clustering analysis presented distinct behavior as to the one observed for the triploid and tetraploid accessions. In the Structure approach, the groups were defined based on the likelihood probability using allelic frequencies that characterize each population [30], making this method more reliable to evaluate the group of individuals. In our study, a limited number of accessions of the distinct subspecies were analyzed (seven accessions of ssp. *malaccensis* at groups I, VII, VIII, XIX; one ssp. *errans* at XVIII; five ssp. *banksii* at group IX; three ssp *burmannica/burmannicoides* at XI, XVIII; four ssp. *siamea* at VII, XI, XVIII; two ssp. *microcarpa* at XI, XVIII; and three ssp. *zebrina* at XI, XVIII), which limit inferences about the relationships among these distinct subspecies. Further, some of these AA diploids can intercross, and the classification in subspecies was merely based on spatial and temporal isolation, and some of the accessions might have an inter-subspecifc origin [2].

Despite the limited number of accessions for each subspecies, inferences from previous studies were supported. For instance, the grouping of five ssp. *banksii* (group IX) accessions with cultivated diploids have been reported [2,84] with a clear distinction from other subspecies [84]. *Musa acuminata* ssp. *banksii* originated in Papua New Guinea and the Northern Indonesian islands, geographically isolated from the other subspecies, and it is a preferential autogamous [2]. Accession of this subspecies, presented low average heterozygosis (55.8%) and PIC value (36.6%). These homozygous loci for *banksii* and the cultivated diploids were also reported by Grapin et al. [73]. When compared with the other subspecies, *banksii* presented high membership values (Figure 4).

In general, there was a diversified behavior of diploids with accessions of the same subspecies in different groups and/or with different subspecies, as verified for groups XI and XVIII (Figure 4). These two groups contained a few accessions of ssp. *burmannica/burmannicoides*; ssp. *siamea*; ssp. *microcarpa* and ssp. z*ebrina*, corroborating the grouping obtained based on DArT [84], and the closer relationships between ssp. *errans* and ssp. *microcarpa*[73]. However, these subspecies demonstrated distinct cytotypes based on PCR-RFLP [85]. Assembling the distinct subspecies into the same cluster has been reported [2,9,84]. This behavior could be associated with the broad variability that exists within *M. acuminata*[91] or the presence of many rare alleles in the subspecies [73] that may obscure genetic relationships. Further, differences in markers and methods of analysis, together with distinct accession names [76], and the identification of some accessions as being from a determined subspecies is still questionable [2] makes direct comparison between studies difficult.

## Conclusions

The *ex situ* collection at ‘Embrapa Mandioca Fruticultura’ Center represents an important source of *Musa* spp. genetic resources. The accessions are characterized according to their agronomic traits, and they have been screened for disease resistance to Black- and Yellow-Sigatoka, *Fusarium* wilt and Moko, and now their ploidy, genomic constitution and genetic diversity have been established. This study represents an initial effort to define genetic relationships within *Musa* using Bayesian statistics implemented in Structure, while exploring the co-dominant nature of microsatellites, not previously performed in *Musa*.

DNA content was believed to be a good predictor of genomic constitution in *Musa*, but our results confirmed that these small differences are potentially overlapped by the occurrence of homeologue recombination, discrepancies in the number of sets or portions from each parental genome, including aneuploidy. Similarly, detection of unexpected ITS rDNA alleles corroborated the hypothesis about the occurrence of recombination between the A and B genomes or between *M. acuminata* subspecies genomes. The occurrence of these phenomenons has been largely disregarded in the evolution of banana cultivars, as the “single-step domestication” hypothesis had long predominated, and these findings will have an impact in future breeding approaches.

Structure analysis enabled the efficient detection of ancestry of recently developed tetraploid hybrids by breeding programs, and for some triploid cultivars. However, for the main commercial subgroups, Structure appeared to be less efficient to detect the ancestry in diploid groups, possibly either due to diploid under-representation in the collection; limited number of analyzed loci evaluated; or allelic changes during evolution of the subgroups, especially the allopolyploids.

Establishing ancestry and genetic relationships by Structure allowed the identification of diploids from group IX and ‘Lareina BT100’ as potentially related to parentals of the sterile ‘Cavendish’ and ‘Gros Michel’ accessions, which could be used in crossing programs or chromosome manipulations (doubling) to obtain/re-synthesize ‘Gros Michel’/‘Cavendish’ hybrids. The possibility of inferring the membership of the accessions using Bayesian analysis opens possibilities for its use in marker-assisted selection by association mapping by incorporating the effects of the structure (matrix of the membership; *q* matriz) in the population to control false positives (type I error) [35,92].

With the completion of the *Musa* genome sequencing [93], together with the development of next-generation sequencing technology, increasing the precision of genomic information will enable an improved definition of the relationships among cultivated bananas and its diploids parental. The evaluation of a larger number of diploid accessions from the various subspecies would allow a better definition of the relationships among diploids and among triploid cultivars, therefore, to use this approach to assist and develop new strategies in breeding programs.

## Competing interests

The authors declare that they have no competing interests.

## Authors’ contributions

ONJ, SOS and AF conceived the study, which was the Doctoral project of ONJ. SOS, EPA and CFF maintained and provided material from the *ex situ Musa* collection, and participated in the interpretation of the data. ONJ and GGS conducted the molecular analyses. JMCS conducted the flow cytometry analyses. ONJ and AF discussed the results and wrote the manuscript with the help of CFF. All authors read and approved the final manuscript.

## Supplementary Material

Additional file 1: Table S1 *Musa* accessions from the *ex situ* collection of ‘Embrapa Mandioca Fruticultura’ Center (Cruz das Almas, Brazil) with original provenance and information on ploidy and genomic composition derived from morphological characterization or information from origin (passport data). **Table S2.** Loci used for the characterization of the *ex situ Musa* collection from ‘Embrapa Mandioca Fruticultura’ Center, containing a tail for fluorescent labeling, with number of observed alleles (Na), Polymorphic Information Content (PIC), Marker Index (MI). Underlined regions refer to tail used to label products with fluorescence FAM, HEX, or NED. **Figure S1.** Mean observed heterozigosity (H_o_) and Polymorphic Information Content (PIC) for all microsatellite loci. C: cultivated; W: wild. **Figure S2.** Histogram representing the proportion (Y-axis) of dissimilarity (X-axis) between pairs of accessions, for all accessions (General) and main genomic groups. Click here for file
