# Supplementary material for: Genetic diversity and population structure of Musa accessions in ex situ conservation
Source: BMC Plant Biol. 2013 Mar 12;13:41. doi: 10.1186/1471-2229-13-41 (PMC3636076; doi:10.1186/1471-2229-13-41)
Supplement: Additional file 1: Table S1 — Musa accessions from the ex situ collection of ‘Embrapa Mandioca Fruticultura’ Center (Cruz das Almas, Brazil) with original provenance and information on ploidy and genomic composition derived from morphological characterization or information from origin (passport data). Table S2. Loci used for the characterization of the ex situ Musa collection from ‘Embrapa Mandioca Fruticultura’ Center, containing a tail for fluorescent labeling, with number of observed alleles (Na), Polymorphic Information Content (PIC), Marker Index (MI). Underlined regions refer to tail used to label products with fluorescence FAM, HEX, or NED. Figure S1. Mean observed heterozigosity (Ho) and Polymorphic Information Content (PIC) for all microsatellite loci. C: cultivated; W: wild. Figure S2. Histogram representing the proportion (Y-axis) of dissimilarity (X-axis) between pairs of accessions, for all accessions (General) and main genomic groups. [file 1471-2229-13-41-S1.docx]

Additional File 1: Table S1. *Musa* accessions from the *ex situ* collection of ‘Embrapa Mandioca Fruticultura’ Center (Cruz das Almas, Brazil) used for ploidy and genomic constitution determination based on flow cytometry, PCR-RFLP of Internal Transcribed Spacer (ITS) regions, and Simple Sequence Region (SSR) loci, with original provenance.Information on ploidy and genomic composition derived from morphological characterization or information from origin (passport data).

| No. | Acession | | | | | | Genome^y^ | Subgroup/Subspecies^z^ | | | | Provenance^y^ |  |
| --- | --- | --- | --- | --- | --- | --- | --- | --- | --- | --- | --- | --- | --- |
| 1 | *Musa basjoo* | | | | | | *Musa* | *-* | | | | France |  |
| 2 | Piraí | | | | | | BB | *-* | | | | Brazil |  |
| 3 | Butuhan | | | | | | BB | *balbisiana* | | | | Phillipines |  |
| 4 | BB Panamá | | | | | | BB | *balbisiana* | | | | Panama |  |
| 5 | Balbisiana França | | | | | | BB | *balbisiana* | | | | France |  |
| 6 | *Musa balbisiana* | | | | | | BB | *balbisiana* | | | | Brazil |  |
| 7 | TIP | | | | | | ABB | *-* | | | | Thailand |  |
| 8 | Saba Honduras | | | | | | ABB | Saba | | | | Honduras |  |
| 9 | Saba | | | | | | ABB | Saba | | | | Costa Rica |  |
| 10 | Prata Zulu | | | | | | ABB | *-* | | | | Brazil |  |
| 11 | Poteau Nain | | | | | | ABB | Bluggoe | | | | France |  |
| 12 | Pelipita | | | | | | ABB | Pelipita | | | | Honduras |  |
| 13 | Namwa Khom | | | | | | ABB | Pisang awak | | | | Thailand |  |
| 14 | Namwa Daeng | | | | | | ABB | *-* | | | | Thailand |  |
| 15 | Muisa Tia | | | | | | ABB | Pisang awak | | | | France |  |
| 16 | Monthan | | | | | | ABB | *-* | | | | France |  |
| 17 | Ice Cream | | | | | | ABB | *-* | | | | Hawaii |  |
| 18 | Ice Cream | | | | | | ABB | *-* | | | | France |  |
| 19 | Gia Hui | | | | | | ABB | Pisang awak | | | | France |  |
| 20 | Figo Cinza | | | | | | ABB | *-* | | | | Brazil |  |
| 21 | Espermo | | | | | | ABB | Bluggoe | | | | Colombia |  |
| 22 | Champa Madras | | | | | | ABB | Bluggoe | | | | France |  |
| 23 | Cachaco | | | | | | ABB | Bluggoe | | | | Colombia |  |
| 24 | Cacambou Naine | | | | | | ABB | Bluggoe | | | | Ecuador |  |
| 25 | Benedetta | | | | | | ABB | Saba | | | | France |  |
| 26 | Abuperak | | | | | | ABB | *-* | | | | France |  |
| 27 | IAC | | | | | | AB(H) | *-* | | | | Brazil |  |
| 28 | Yangambi nº2 | | | | | | AAB | Silk | | | | France |  |
| 29 | Warik | | | | | | AAB | *-* | | | | New Guinea |  |
| 30 | Walha | | | | | | AAB | Pome | | | | Hawaii |  |
| 31 | Ustrali | | | | | | AAB | *-* | | | | Indonesia |  |
| 32 | Umpako | | | | | | AAB | *-* | | | | New Guinea |  |
| 33 | Thap Maeo | | | | | | AAB | *-* | | | | Brazil |  |
| 34 | Trois Vert | | | | | | AAB | *-* | | | | France |  |
| 35 | Tomnam | | | | | | AAB | *-* | | | | New Guinea |  |
| 36 | Tipo Velhaca | | | | | AAB | | | *-* | | | Brazil | |
| 37 | Tip Kham | | | | | AAB | | | *-* | | | Thailand | |
| 38 | Thong Ruong | | | | | AAB | | | *-* | | | Thailand | |
| 39 | Terrinha | | | | | AAB | | | Plantain | | | Brazil | |
| 40 | Terra S/ Nome | | | | | AAB | | | Plantain | | | Brazil | |
| 41 | Tai | | | | | ABB | | | *-* | | | Thailand | |
| 42 | Sempre Verde | | | | | AAB | | | *-* | | | Brazil | |
| 43 | Saney | | | | | AAB | | | *-* | | | New Guinea | |
| 44 | Samurá B | | | | | AAB | | | *-* | | | Brazil | |
| 45 | Red Yade | | | | | AAB | | | Plantain | | | France | |
| 46 | Pulut | | | | | AAB | | | *-* | | | Indonesia | |
| 47 | Pratão | | | | | AAB | | | Pome | | | Brazil | |
| 48 | Prata Sta. Maria | | | | | AAB | | | Pome | | | Brazil | |
| 49 | Prata Ponta Aparada | | | | | AAB | | | Pome | | | Brazil | |
| 50 | Prata Maceió | | | | | AAB | | | Pome | | | Brazil | |
| 51 | Prata Comum | | | | | AAB | | | Pome | | | Brazil | |
| 52 | Prata IAC | | | | | AAB | | | Pome | | |  | |
| 53 | Prata Anã | | | | | AAB | | | Pome | | | Brazil | |
| 54 | Prata Branca | | | | | AAB | | | Pome | | |  | |
| 55 | Poovan | | | | | AAB | | | Silk/Mysore | | | Hawaii | |
| 56 | Plantain N. 2 | | | | | AAB | | | Plantain | | | France | |
| 57 | Pinha | | | | | AAB | | | *-* | | | Brazil | |
| 58 | Padath | | | | AAB | | | | | *-* | | Brazil |  |
| 59 | Pacovan | | | | AAB | | | | | Pome | | Brazil |  |
| 60 | N. 113 | | | | AAB | | | | | *-* | | Thailand |  |
| 61 | Mysore | | | | AAB | | | | | Mysore | | Brazil |  |
| 62 | Muracho | | | | AAB | | | | | *-* | | Belgium |  |
| 63 | Mongolo | | | | AAB | | | | | *-* | | Brazil |  |
| 64 | Moenang | | | | AAB | | | | | *-* | | Thailand |  |
| 65 | Maçã Caule Roxo | | | | AAB | | | | | *-* | | Brazil |  |
| 66 | Kune | | | | AAB | | | | | *-* | | New Guinea |  |
| 67 | Kingala N.1 | | | | AAB | | | | | Silk | | France |  |
| 68 | Kepok Bung | | | | AAB | | | | | *-* | | Indonesia |  |
| 69 | Kelat | | | | AAB | | | | | *-* | | France |  |
| 70 | Java IAC | | | | AAB | | | | | *-* | | Brazil |  |
| 71 | Garoto | | | | AAB | | | | | *-* | | New Guinea |  |
| 72 | Figue Rose Naine | | | | | | AAB | | | | Red | France |  |
| 73 | Eslesno | | | | | | AAB | | | | *-* | Hawaii |  |
| 74 | Curare Enano | | | | | | AAB | | | | Plantain | Costa Rica |  |
| 75 | Comprida | | | | | | AAB | | | | *-* | Brazil |  |
| 76 | Chifre De Vaca | | | | | | AAB | | | | *-* | Brazil |  |
| 77 | Adimoo | | | | | | AAB | | | | *-* | New Guinea |  |
| 78 | AAB S/Nome | | | | | | AAB | | | | *-* | Honduras |  |
| 79 | BRS Tropical | | | | | | AAAB | | | | (Yangambi nº 2 x M53)^x^ | Brazil |  |
| 80 | Preciosa | | | | | | AAAB | | | | (Pacovan x M53)^x^ | Brazil |  |
| 81 | Porp | | | | | | AAAB | | | | *-* | New Guinea |  |
| 82 | Platina | | | | | | AAAB | | | | *-* | Brazil |  |
| 83 | Pacova Ken | | | | | | AAAB | | | | (Pacovan x M53)^x^ | Brazil |  |
| 84 | BRS Platina | | | | | | AAAB | | | | (Prata Anã x M53)^x^ | Brazil |  |
| 85 | Ouro Da Mata | | | | | | AAAB | | | | Pome | Brazil |  |
| 86 | Ngern | | | | | | AAAB | | | | *-* | Thailand |  |
| 87 | Langka | | | | | | AAAB | | | | *-* | Thailand |  |
| 88 | Garantida | | | | | | AAAB | | | | (Prata São Tomé x M53)^x^ | Brazil |  |
| 89 | FHIA-21 | | | | | | AAAB | | | | Plantain Hybrid^w^ | Honduras |  |
| 90 | FHIA-18 | | | | | | AAAB | | | | (Prata Anã x SH3142)^w^ | Honduras |  |
| 91 | FHIA-02 | | | | | | AAAB/AAAA | | | | (Williams x SH3393)^w^ | Honduras |  |
| 92 | FHIA-01 | | | | | | AAAB | | | | (Prata Anã x SH3142)^w^ | Honduras |  |
| 93 | IC - 2 | | | | | | AAAA | | | | (Highgate x 2*n*) | Brazil |  |
| 94 | Calypso | | | | | | AAAA | | | | (Highgate x 2*n*) | Santa Lucia |  |
| 95 | Buccaneer/Bucaneiro | | | | | | AAAA | | | | (Highgate x 2*n*) | Santa Lucia |  |
| 96 | Ambrosia | AAAA | | | | | | (Highgate x 2*n*) | | | | Santa Lucia |  |
| 97 | Yangambi Km 5 | AAA | | | | | | Ibota | | | | France |  |
| 98 | Wasolay | AAA | | | | | | *-* | | | | New Guinea |  |
| 99 | Walebo | AAA | | | | | | *-* | | | | New Guinea |  |
| 100 | Valery | AAA | | | | | | Cavendish | | | | Brazil |  |
| 101 | Umbuk | AAA | | | | | | *-* | | | | Indonesia |  |
| 102 | Tugoomomboo | AAA | | | | | | *-* | | | | New Guinea |  |
| 103 | Caipira | AAA | | | | | | Ibota | | | | Brazil |  |
| 104 | Towoolee | AAA | | | | | | *-* | | | | New Guinea |  |
| 105 | Torp | AAA | | | | | | *-* | | | | New Guinea |  |
| 106 | Sri | AAA | | | | | | *-* | | | | Indonesia |  |
| 107 | Sapon | | | AAA | | | | *-* | | | | Indonesia |  |
| 108 | São Tomé | | | AAA | | | | *-* | | | | Brazil |  |
| 109 | Roombum | | | AAA | | | | *-* | | | | New Guinea |  |
| 110 | Poyo | | | AAA | | | | Cavendish | | | | Brazil |  |
| 111 | Pirua | | | AAA | | | | *-* | | | | Brazil |  |
| 112 | Pagatow | | | AAA | | | | *-* | | | | New Guinea |  |
| 113 | Ouro Mel | | | AAA | | | | *-* | | | | Brazil |  |
| 114 | Orotawa | | | AAA | | | | *-* | | | | France |  |
| 115 | Nanicão | | | AAA | | | | Cavendish | | | | Brazil |  |
| 116 | Nam | | | AAA | | | | *-* | | | | Thailand |  |
| 117 | Muga | | | AAA | | | | *-* | | | | New Guinea |  |
| 118 | Morong | | | AAA | | | | *-* | | | | New Guinea |  |
| 119 | Markatooa | | | AAA | | | | *-* | | | | New Guinea |  |
| 120 | Maida | | | AAA | | | | *-* | | | | Costa Rica |  |
| 121 | Leite | | | AAA | | | | *-* | | | | Brazil |  |
| 122 | Lacatan | | | AAA | | | | Cavendish | | | | Brazil |  |
| 123 | Azedinha | | | AAB | | | | *-* | | | | Brazil |  |
| 124 | Imperial | | | AAA | | | | *-* | | | | Brazil |  |
| 125 | Highgate | | | AAA | | | | *-* | | | | Honduras |  |
| 126 | Gros Michel | | | AAA | | | | Gros Michel | | | | Brazil |  |
| 127 | Grande Naine | | | AAA | | | | Cavendish | | | | Brazil |  |
| 128 | Dois Cachos | | | AAA | | | | *-* | | | | Brazil |  |
| 129 | Dodoga | | | AAA | | | | *-* | | | | New Guinea |  |
| 130 | Cocos | | | AAA | | | |  | | | | France |  |
| 131 | Caru Verde | | | AAA | | | | Red | | | | Brazil |  |
| 132 | Caru Roxa | | | AAA | | | | Red | | | | Brazil |  |
| 133 | Canela | | | AAA | | | | *-* | | | | Brazil |  |
| 134 | Bakar | | AAA | | | | | *-* | | | | Indonesia (FHIA) |  |
| 135 | Bagul | | AAA | | | | | *-* | | | | New Guinea |  |
| 136 | Amritsagar | | AAA | | | | | *-* | | | | Havaí |  |
| 137 | Ambei | | AAA | | | | | *-* | | | | New Guinea |  |
| 138 | AAA Desconhecida | | AAA | | | | | *-* | | | | New Guinea |  |
| 139 | Zebrina | | AA(W) | | | | | ssp*. zebrina* | | | | Hawaii |  |
| 140 | Selangor | | AA(W) | | | | | ssp*. malaccensis* | | | | France |  |
| 141 | Perak | | AA(W) | | | | | *-* | | | | Malaysia |  |
| 142 | Pa Songkla | | AA(W) | | | | | ssp*. siamea* | | | | France |  |
| 143 | Pa Rayong | | AA(W) | | | | | ssp*. siamea* | | | | Thailand |  |
| 144 | Pa Phatthalung | | | | | AA(W) | | *-* | | | | Thailand |  |
| 145 | Pa Musore 3 | | | | | AA(W) | | ssp*. malaccensis* derived | | | | Thailand |  |
| 146 | Pa Musore 2 | | | | | AA(W) | | ssp*. malaccensis* derived | | | | Thailand |  |
| 147 | Pa Abissinea | | | | | AA(W) | | ssp*. siamea* | | | | Thailand |  |
| 148 | N.118 | | | | | AA(W) | | *-* | | | | Thailand |  |
| 149 | Monyet | | | | | AA(W) | | ssp*. zebrina* | | | | Indonesia |  |
| 150 | Modok Gier | | | | | AA(W) | | *-* | | | | New Guinea |  |
| 151 | Microcarpa | | | | | AA(W) | | ssp*. microcarpa* | | | | France |  |
| 152 | Malaccensis | | | | | AA(W) | | ssp*. malaccensis* | | | | Honduras |  |
| 153 | Krasan Saichon | | | | | AA(W) | | *-* | | | | Thailand |  |
| 154 | Khae | | | | | AA(W) | | *-* | | | | Thailand |  |
| 155 | Jambi | | | | | AA(W) | | ssp*. malaccensis* | | | | Indonesia |  |
| 156 | Cici | | | | | AA(W) | | ssp*. malaccensis* | | | | Indonesia |  |
| 157 | Calcutta 4 | | | | | AA(W) | | ssp*. burmanica/burmannicoide* | | | | Jamaica |  |
| 158 | Burmannica | | | | | AA(W) | | ssp*. burmanica/burmannicoide* | | | | Honduras |  |
| 159 | Buintenzorg | | | | | AA(W) | | ssp*. zebrina* | | | | Jamiaca |  |
| 160 | Birmanie | | | | | AA(W) | | *-* | | | | France |  |
| 161 | M 61 | | | | | AA(H) | | Hybrid | | | | Ecuador |  |
| 162 | M 53 | | | | | AA(H) | | Hybrid | | | | Ecuador |  |
| 163 | M 48 | | | | | AA(H) | | Hybrid | | | | Ecuador |  |
| 164 | F3P4 | | | | | AA(H) | | Hybrid | | | | Ecuador |  |
| 165 | F2P2 | | | | | AA(H) | | Hybrid | | | | Ecuador |  |
| 166 | Tuugia | | | | | AA(C) | | *-* | | | | Hawaii |  |
| 167 | Tongat | | | | | AA(C) | | ssp*. errans* | | | | Honduras |  |
| 168 | Giral | | | | | AAB | | *-* | | | | Brazil |  |
| 169 | Tjau Lagada | | | | | AA(C) | | ssp*. microcarpa* derived | | | | Honduras |  |
| 170 | Thong Dok Mak | | | | | AA(C) | | *-* | | | | Thailand |  |
| 171 | TA | | | | | AA(C) | | *-* | | | | New Guinea |  |
| 172 | Sowmuk | | | | | AA(C) | | ssp*. banksii* | | | | New Guinea |  |
| 173 | SA | | | | | AA(C) | | *-* | | | | Thailand |  |
| 174 | S/N. 2 | | | | | AA(C) | | ssp*. banksii* | | | | New Guinea |  |
| 175 | Raja Uter | | | | | AA(C) | | *-* | | | | Indonesia |  |
| 176 | Pipit | | | | | AA(C) | | *-* | | | | Indonesia |  |
| 177 | Ouro | | | | | AA(C) | | *-* | | | | Brazil |  |
| 178 | Niyarma Yik | | | | | AA(C) | | ssp*. banksii* | | | | Thailand |  |
| 179 | NBF 9 | | | | | AA(C) | | *-* | | | | New Guinea |  |
| 180 | NBA 14 | | | | | AA(C) | | ssp*. banksii* | | | | New Guinea |  |
| 181 | Mangana | | | | | AA(C) | | *-* | | | | New Guinea |  |
| 182 | Mambee Thu | | | | | AA(C) | | ssp*. banksii* | | | | New Guinea |  |
| 183 | Malbut | | | | | AA(C) | | *-* | | | | New Guinea |  |
| 184 | Lidi | | | | | AA(C) | | *-* | | | | Honduras |  |
| 185 | Khi Maeo | | | | | AA(C) | | *-* | | | | Thailand |  |
| 186 | Khai Nai On | | | | | AA(C) | | *-* | | | | Thailand |  |
| 187 | Khai | | | | | AA(C) | | *-* | | | | Thailand |  |
| 188 | Jari Buaya | | | | | AA(C) | | *-* | | | | Honduras |  |
| 189 | Jaran | | | | | AA(C) | | ssp.*burmanica/burmannicoide* | | | | Indonesia |  |
| 190 | Fako Fako | | | | | AA(C) | | *-* | | | | New Guinea |  |
| 191 | Berlin | | | | | AA(C) | | *-* | | | | Indonesia |  |
| 192 | Babi Yadefana | | | | | AA(C) | | *-* | | | | New Guinea |  |
| 193 | Prata Manteiga | | | | | AAB | | Pome | | | | Brazil |  |
| 194 | Borneo | | | | | AA (W) | | ssp. *microcarpa* | | | | Jamaica |  |
| 195 | Madu | | | | | AA | | *-* | | | | Honduras |  |
| 196 | Prata Maçã | | | | | AAAB | | Pome | | | | Brazil |  |
| 197 | Verde | | | | | AAB | | *-* | | | | Brazil |  |
| 198 | Prata Anã 2 | | | | | AAB | | Pome | | | | Brazil |  |
| 199 | Prata Anã 3 | | | | | AAB | | Pome | | | | Brazil |  |
| 200 | Pacovan Ken | | | | | AAAB | | (Pacovan x M53)^x^ | | | | Brazil |  |
| 201 | Pitogo | | | | | ABB | | *-* | | | | Brazil |  |
| 202 | Pacha Nadan | | | | | AB | | *-* | | | | Brazil |  |
| 203 | Njok Kon | | | | | AAB | | *-* | | | | France |  |
| 204 | Marmelo | | | | | NI | | *-* | | | | Brazil |  |
| 205 | Lareina BT100 | | | | | NI | | *-* | | | | - |  |
| 206 | Pisang Ceylan | | | | | AAB | | *-* | | | | Belgium |  |
| 207 | Pisang Nangka | | | | | AA | | *-* | | | | Brazil |  |
| 208 | Willians | | | | | AAA | | Cavendish | | | | Belgium |  |
| 209 | PV42-114 | | | | | AAAB | | (Pacovan x M53)^x^ | | | | Brazil |  |
| 210 | PV03-76 | | | | | AAAB | | | (Pacovan x Calcutta 4)^w^ | | | Brazil | |
| 211 | Khae Prae | | | | | AA | | | ssp*. siamea* | | |  | |
| 212 | Pitu | | | | | AA | | | *-* | | |  | |
| 213 | Paka Iv | | | | | AA | | | *-* | | |  | |
| 214 | Ido 110 | | | | | AA | | | *-* | | |  | |
| 215 | P. Kermain | | | | | NI | | | *-* | | |  | |
| 216 | P. Serum | | | | | AA | | | ssp*.malaccensis* | | |  | |
| 217 | Pisang Mas | | | | | AA | | | *-* | | |  | |
| 218 | Uwati | | | | | AA | | | *-* | | |  | |
| 219 | Diplóide Bélgica | | | | | AA | | | *balbisiana* | | | Belgium | |
| 220 | BB France | | | | | BB | | | *balbisiana* | | | France | |
| 221 | BB IAC | | | | | BB | | | *balbisiana* | | | Brazil | |
| 222 | *Musa laterita* | | | | | *Musa* | | | *Rhodochlamys* | | | France | |
| 223 | Tambi | | | | | AAA | | | *-* | | | Honduras | |
| 224 | Royal (*M. ornata* x *M. velutina*) | | | | | *Musa* | | | *Rhodochlamys* | | | France | |

^z^Information based on the *Musa* MGIS database [46];

^y^NI – no information available;

^x^Information from passport data from ‘Embrapa Mandioca Fruticultura’ - *Musa* = distinct species from *M. acuminata* or *M. balbisiana*; C=cultivar; W = wild; H = Hybrid; NI = no information;

^x^Hybrid developed by ‘Embrapa Mandioca Fruticultura’;

^w^Hybridsdeveloped by FHIA (*Fundación Hondureña de Investigación Agrícola*).

Additional File 2: Table S2. Loci used for the characterization of the *exsituMusa* collection from ‘Embrapa Mandioca Fruticultura’ Center, containing a tail for fluorescent labeling, with number of observed alleles (Na), Polymorphic Information Content (PIC), Marker Index (MI). Underlined regions refer to tail used to label products with fluorescence FAM, HEX, or NED.

| **Locus** | **Forward (5'-3')** | **Reverse (5'-3')** | ***Na*** | **PIC** | **MI** |
| --- | --- | --- | --- | --- | --- |
| *Ma1-17* | gactatgggcgtgagtgcattgaggcggggaatcggta | ggcgggagacagatggagtt | 11 | 0.23 | 2.52 |
| *Ma1-27* | gactatgggcgtgagtgcattgaatcccaagtttggtcaag | caaaacactgtccccatctc | 10 | 0.18 | 1.76 |
| *AGMI 93/94* | gactatgggcgtgagtgcataacaactaggatggtaatgtgtgga | gatctgaggatggttctgttggag | 15 | 0.16 | 2.41 |
| *AGMI 67/68* | gactatgggcgtgagtgcatataccttctcccgttcttcttc | tggaaacccaatcattgatc | 10 | 0.18 | 1.84 |
| *Mb1–100* | ggctaggaaaggttagtggctcggctggctaatagaggaa | tctcgagggatggtgaaaga | 10 | 0.21 | 2.09 |
| *MaOCEN01* | accaacctaggaaacacagttctcaggaagggcaacaatc | ggaccaaagggaaagaaacc | 13 | 0.20 | 2.57 |
| *MaOCEN03* | accaacctaggaaacacagtggaggaaatggaggtcaaca | ttcgggataggaggaggag | 9 | 0.17 | 1.57 |
| *MaOCEN10* | ggctaggaaaggttagtggcggaagaaagaagtggagaatgaa | tgaaatggataaggcagaagaa | 14 | 0.17 | 2.40 |
| *MaOCEN12* | accaacctaggaaacacagtgcaagaaagaacgagaaggaaa | gtggggagggaggcatag | 8 | 0.26 | 2.07 |
| *MaOCEN13* | gactatgggcgtgagtgcatgctgctattttgtccttggtg | cttgatgctgggattctgg | 11 | 0.18 | 1.97 |
| *MaOCEN14* | ggctaggaaaggttagtggctcttttgcgtgagtttttgg | cgtgggaggaacagtgaa | 14 | 0.21 | 2.96 |
| *MaOCEN19* | ggctaggaaaggttagtggccgaagagcaccacaaagga | gcagcagaagcaagatagca | 15 | 0.16 | 2.42 |
| *MaO-EC02***^Z^** | ggctaggaaaggttagtggcggggaaggtggtgtagga | ggcaaatggaagagaggag | 10 | 0.30 | 2.98 |
| *MaO-FA12***^Z^** | accaacctaggaaacacagtcccgtgtattggtgtcgtag | gagactgatggcaaaggatg | 7 | 0.26 | 1.83 |
| *MaC-CEN04***^Z^** | gactatgggcgtgagtgcattgtcagataggtcggagttg | agtgctcttgttaggtttcc | 15 | 0.22 | 3.24 |
| *MaC-CEN06***^Z^** | accaacctaggaaacacagtttctgctgggctgtctatga | aagggcagttcacaacacaa | 12 | 0.16 | 1.92 |
| **Mean** |  |  | **11.5** | **0.20** | **2.28** |

^Z^Loci *MaO-EC02*, *MaO-FA12*, *MaC-CEN04*, and *MaC-CEN06* have not been published.

Additional File 3: Figure S1. Mean observed heterozigosity (H_o_) and Polymorphic Information Content (PIC) for all microsatellite loci.C: cultivated; W: wild.


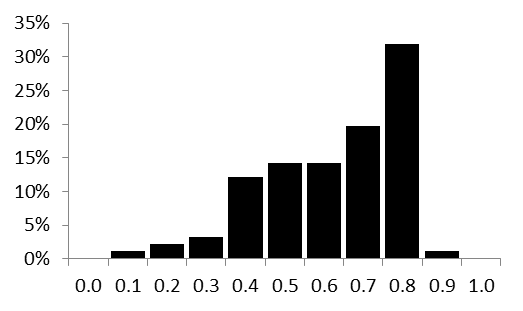

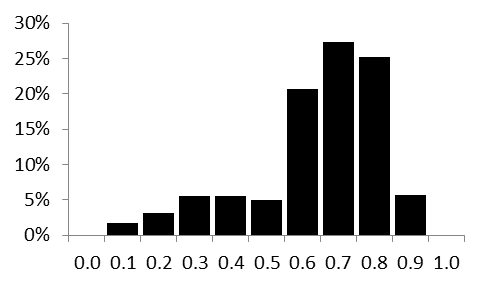

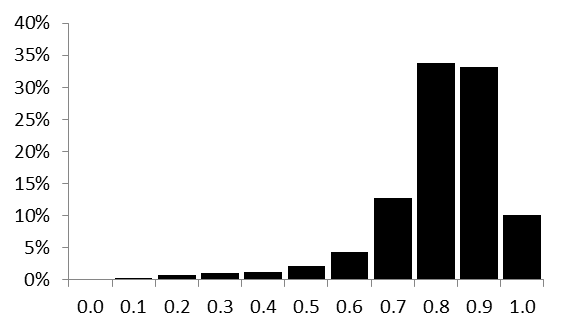

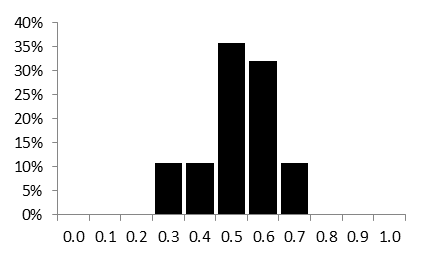

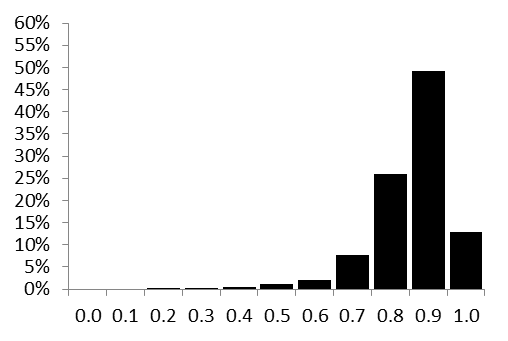

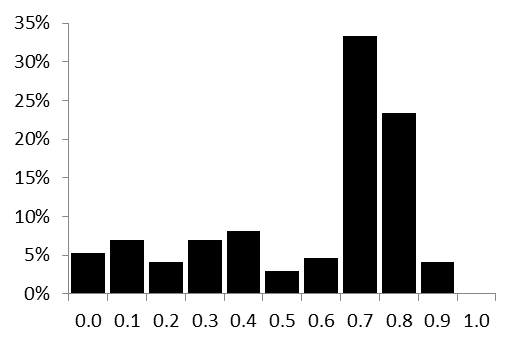

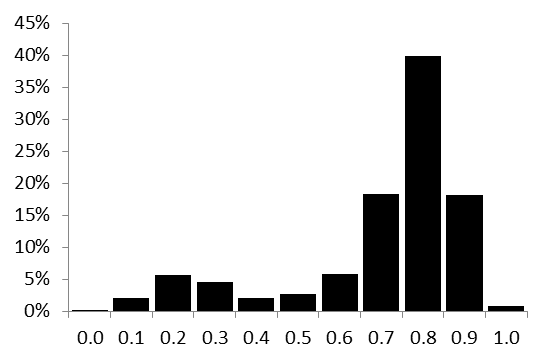


General

AA

BB

ABB

AAB

AAAB

AAA

Additional File 4: Figure S2. Histogram representing the proportion (Y-axis) of dissimilarity (X-axis) between pairs of accessions, for all accessions (General) and main genomic groups.
